# High-throughput conjugation reveals strain specific recombination patterns enabling precise trait mapping in *Escherichia coli*

**DOI:** 10.1371/journal.pgen.1011636

**Published:** 2025-10-30

**Authors:** Thibault Corneloup, Juliette Bellengier, Isabelle Rosinski-Chupin, Mélanie Magnan, Arsh Chavan, Benoit Gachet, Zoya Dixit, Coralie Pintard, Alexandra Baron, Doreen Toko, Amaury Lambert, Alaksh Choudhury, Olivier Tenaillon

**Affiliations:** 1 Université Paris Cité and Université Sorbonne Paris Nord, Inserm, IAME, Paris, France; 2 Université Paris Cité, CNRS, Inserm, Institut Cochin, Paris, France; 3 Université Paris Cité, Paris, France; 4 Ecology and Evolution of Antibiotic Resistance Unit, Institut Pasteur, Paris, France; 5 Department of Biology, Indian Institute of Science Education and Research, Pune, India; 6 Centre de Recherche sur l’Inflammation, INSERM U1149, Université Paris Cité, Paris, France; 7 Laboratoire de Biophysique et Evolution (LBE), ESPCI, UMR CNRS-ESPCI 8231 Chemistry, Biology and Innovation, Paris, France; 8 Center for Interdisciplinary Research in Biology (CIRB), Collège de France, CNRS, INSERM, Université PSL, Paris, France; 9 Institut de Biologie de l’ENS (IBENS), École Normale Supérieure (ENS), CNRS, INSERM, Université PSL, Paris, France; 10 Génomique Métabolique, Genoscope, Institut François Jacob, CEA, CNRS, Univ Evry, Université Paris-Saclay, Evry-Courcouronnes, France; Indiana University Bloomington, UNITED STATES OF AMERICA

## Abstract

Genetic exchange is a cornerstone of evolutionary biology and genomics, driving adaptation and enabling the identification of genetic determinants underlying phenotypic traits. In *Escherichia coli*, horizontal gene transfer via conjugation and transduction not only promotes diversification and adaptation but has also been instrumental in mapping genetic traits. However, the dynamics and variability of bacterial recombination remain poorly understood, particularly concerning the patterns of recombined DNA fragments. To elucidate these patterns and simultaneously develop a tool for trait mapping, we designed a high-throughput conjugation method to generate recombinant libraries. Recombination profiles were inferred through whole-genome sequencing of individual clones and populations after selection of a marker from the donor strain in the recipient. This analysis revealed an extraordinary range of recombined fragment sizes, spanning less than ten kilobases to over a megabase—a pattern that varied across the three tested strains. Mathematical modelling indicated that this diversity in recombined fragment size enables precise identification of selected loci following genetic crosses. Consistently, population sequencing pinpointed a selected marker at kilobase-scale accuracy, offering a robust tool for identifying subtle genetic determinants that could include point mutations in core genes. These findings challenge the conventional view that conjugation always transfers large fragments, suggesting that even short recombined segments, traditionally attributed to transduction, may originate from conjugation.

## Author summary

*Escherichia coli* demonstrates a remarkable ability to adapt to diverse environments. This adaptability is in part connected to its ability to recombine incoming DNA in its chromosome, as illustrated by the vast gene content diversity observed in the species. However, the precise dynamics and patterns of bacterial recombination remain poorly understood. To address this knowledge gap, we developed a high-throughput method to observe genetic transfer in the laboratory based on conjugation. Surprisingly, we found that the size of transferred DNA fragments varied from less than 10 kilobases up to a megabase. These results challenge the long-held belief that conjugation primarily transfers only large DNA segments. This diversity in fragment sizes has important consequences. First as a genetic tool, it enables us to pinpoint the location of specific traits in the genome with gene resolution. Second and more importantly, short fragment recombination allows a specific selected loci to be transferred from one strain to another with minimum collateral material allowing an almost surgical precision in genome evolution. Finally, using three different stains as recipient, we observe a strain specific pattern of recombination suggesting a genetic control of that process that will require further investigation.

## 1. Introduction

Genetic exchanges occupy a pivotal position in genomics, serving two complementary roles. First, by severing the physical linkage between alleles during a population’s evolutionary journey, it ensures that alleles would rise or fall in frequency thanks to their own selective value rather than being affected by the selective impact of their chromosomal neighbours [1]. Second, being able to isolate the effect of a single allele from the surrounding genome is also central to geneticists’ ability to uncover the genetic underpinnings of phenotypic traits. From tracing genealogies to conducting quantitative trait loci (QTL) analyses and genome-wide association studies (GWAS), genetic exchange is crucial for pinpointing chromosomal regions linked to traits.

In bacterial genomes, where reproduction is uncoupled from genetic exchange, these transfers remain no less crucial. Processes such as conjugation, transduction, and natural transformation drive both genome evolution and scientific discovery [2]. These mechanisms facilitate the spread of beneficial alleles or gene clusters, irrespective of their genomic background. Take the High Pathogenicity Island (HPI) in *Escherichia coli*: this genomic region, essential for efficient iron acquisition in diverse host environments, owes its dissemination within the species to homologous recombination following conjugation [3]. Such transfers have not only fostered bacterial adaptation but have also enabled HPI identification as a major virulence determinant using agnostic statistical approaches such as GWAS [4]. In addition, similarly to eukaryotic crosses, conjugation was instrumental in crafting the first bacterial genetic maps by tracking the inheritance of selectable markers [5].

While the mechanics of eukaryotic recombination are well-studied, bacterial recombination remains more enigmatic. For example, conjugation involves double crossovers, resulting in varying integration lengths [6,7]. In contrast, another type of recombination such as natural transformation, may integrate single stranded DNA without requiring double crosses over. Complicating matters further, as incoming DNA is often a threat, bacteria have evolved core stress responses or accumulated accessory defence systems [8], including restriction-modification genes, to degrade incoming DNA. Such defences fragment donor DNA, with the pattern of fragmentation potentially depending on strain genomic content. To better investigate these issues, we have developed a model of conjugation to unravel how conjugation shapes genome evolution and enables functional genomics.

Horizontal gene transfer in *E. coli* drives genomic diversification via two primary mechanisms. One involves phages erroneously packaging host DNA—a process known as transduction—while the other relies on plasmid-mediated conjugation. The relative importance of these mechanisms in nature remains contested. Comparative analyses of closely related genomes suggest that transduction predominates, with recombinant fragments spanning tens of kilobases, matching experimental observations [9,10]. By contrast, conjugation transfers fragments potentially exceeding hundreds of kilobases. Mastery of these two processes has underpinned genetic engineering for decades. Transduction, with its low efficiency, has been most useful for transferring selectable markers across genetic backgrounds. Conjugation, by contrast, has been a workhorse for genetic mapping [5] and, more recently, for assembling complex genome constructs and high-throughput marker transfers [11].

Despite these advances, identifying the genetic basis of phenotypic traits in bacteria remains challenging. In a genetically diverse species like *E. coli*, phenotypic diversity can stem from gene presence or absence, as exemplified by the HPI [3] or antibiotic resistance genes. Yet, over a decade of experimental evolution, coupled with whole-genome sequencing, revealed that single-point mutations in conserved genes or regulatory regions can drive significant adaptive changes [12–14]. Adaptation may even involve mutations in essential genes [15]. Classical tools like transposon mutagenesis, while invaluable for gene inactivation studies, often fail to pinpoint these causative alleles. QTL-style crosses could address this limitation, provided the resulting genetic linkage is minimal.

Multiple experimental designs have been developed recently to study the heterogeneity of transfer in bacterial populations and/or to do trait mapping or simply as tools to modify genomes. For instance, Silbert et al [16] modified the broad host range plasmid RP4 to be integrated directed in the chromosome with a transposase, while Khetrapal et al. [17] integrated the origin of transfer of that plasmid through transposon mutagenesis while providing the plasmid transfer genes in trans. In the latter case, Mass Allelic exchange (MAE) was observed [17] and transfer of a virulent phenotype could be used to identify a 314kb region associated with that trait, which further reduced to a single gene via cloning of candidate operons.

In the present study, we leverage the MAE approach to investigate the recombination patterns and the heterogeneity of recombination fragments across *E. coli* strains, focusing on a specific loci. We developed an alternative experimental design relying on the classical *E. coli* conjugative plasmid F. Our findings revealed that conjugation under identical conditions yields fragment lengths spanning more than two orders of magnitude, with recombination patterns and fragment lengths being strain-specific. This heterogeneity, combined with pool sequencing, enabled precise identification of selected loci following crosses, a result corroborated by mathematical modelling. By shedding light on the extent of bacterial recombination, our approach provides an additional tool for functional genomics and highlights the potentially broader significance of conjugation in shaping *E. coli* genome evolution.

## 2. Results

### 2.1. Rationale for the design of a library of High-frequency Recombination (Hfr) variants

**Fig 1:**
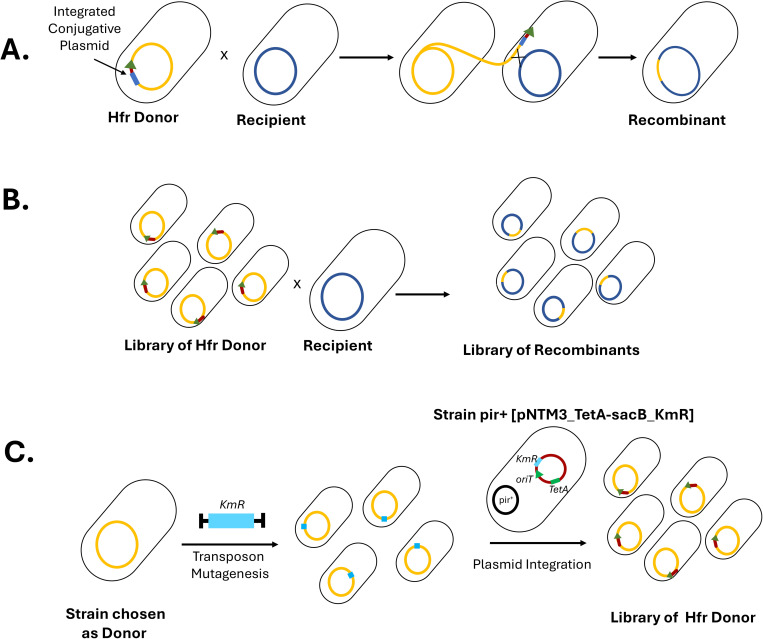
Creation of single-locus Hfr donor versus a multi-locus Hfr donor. A) Chromosomal DNA transfer from a single locus Hfr strain to a recipient showing position-dependent bias in the transferred DNA fragment. The dark blue box and the green arrow represent the integrated conjugative plasmid and its origin of transfer, respectively. B) DNA transfer between a library of Hfr and a single recipient. Here, different fragments are transferred between the donor and the recipient. C) A landing pad, here a Kanamycin cassette, can be integrated at a random position in the genome with a transposon mutagenesis approach based on Mariner or Tn5 transposons. Homology between the cassette on the plasmid and the one on the chromosome promotes plasmid integration at different location generating a library of Hfr Donor.

For more than 70 years, it has been known that the transfer of chromosomal fragments through conjugation can occur at high frequency with a conjugative plasmid inserted in the chromosome [18]. Parts of the chromosome directly adjacent to the integrated conjugative plasmid, 5’ upstream of the origin of transfer (*oriT*), are transferred from the High-frequency recombination (Hfr) bacteria [19] (Fig 1A). As a consequence, conjugation from a single given Hfr to a recipient strain results in a biased transfer of genetic material proximal to the integration site. A major objective for exploiting conjugative recombination is to discover unknown loci imparting novel phenotypic traits in natural isolates of *E. coli* [20]. Since the position of such loci is unknown, a biased transfer proximal to a fixed position in the chromosome may limit their discovery. Therefore, to increase the success of discovery, we need to initiate transfer from multiple positions along the chromosome simultaneously (Fig 1B). To achieve an unbiased and uniform transfer of DNA from a donor strain, rather than using a single Hfr, we decided to create a library of Hfr donors, each having the conjugative plasmid integrated at a different position on the chromosome.

Conjugative plasmid integration occurs mostly through homologous recombination between plasmid and chromosome sequences that are highly similar [19]. The similarity between the plasmid and the chromosome is often the result of insertion sequences (IS), which can easily change their position (jump) within the genome [21]. The distribution of IS is variable between strains, and most IS elements are present in a few copies on the chromosome [22]. Therefore, IS offer only a limited, variable and biased number of insertion sites. We decided to use a conjugative plasmid that has been shortened to be IS-free: pNTM3 [11]. This suicide plasmid was designed to enhance its integration into the chromosome by replacing its native origin of replication with an R6K origin of replication (S1A Fig). This origin requires the presence of the *pir* gene to maintain the plasmid [23]. The *pir* gene is provided in trans in a permissive strain that allows replication. In strains lacking *pir*, the plasmid, encoding an antibiotic resistance gene, can be grown in media with the corresponding antibiotic only if the plasmid has integrated in the genome to maintain the resistance genes. The pNTM3 plasmid has been previously modified by the integration of certain chromosomal fragments to integrate at various sites on the chromosome [20]. However, our goal was to be able to integrate it at random positions in the chromosome of *E. coli*. To achieve this, we decided to introduce a genomic landing pad with homology to the pNTM3 plasmid. We took advantage of the well-characterized transposon mutagenesis technique (Fig 1C) [24]. Using the machinery of a Tn5 transposable element (or Mariner transposon see *Methods*), we can easily introduce a Kanamycin resistance cassette (*KmR*) at random positions of the *E. coli* chromosome. Although not all natural isolates are amenable to genetic manipulation, many strains can be modified using transposon mutagenesis. To use the Kanamycin landing pad for the chromosomal integration of the pNTM3 plasmid, we also introduced a Kanamycin resistance gene (*KmR*) into the plasmid near the origin of replication. We constructed the plasmid pNTM3_TetA-sacB_KmR (S1A Fig). Upon transferring this modified plasmid to a transposon mutagenesis library of variants (with the *KmR* gene randomly integrated at different positions on the genome), we expect to obtain a library of Hfr variants with this conjugative plasmid randomly integrated at different sites across the genome.

### 2.2. Heterogeneous integration rates of the conjugative machinery

**Fig 2:**
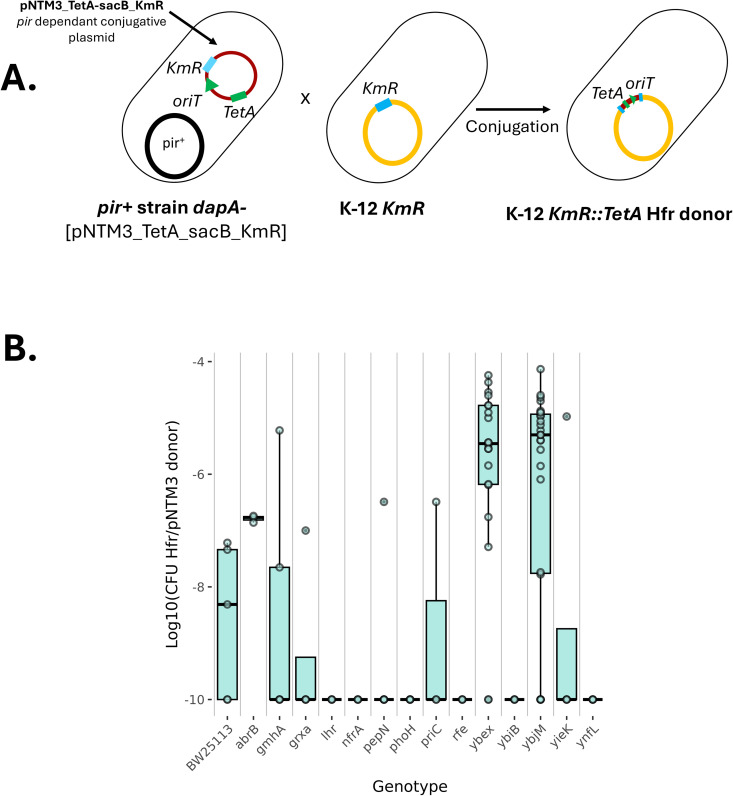
Position-dependent variability in Hfr construction. A) Schematic representation of the conjugation step to create an Hfr strain. The suicide plasmid pNTM3_TetA-sacB_KmR with a tetracyclin resistance (*tetA*) and *KmR* as the homologous marker for integration into the recipient genome, is replicated into a pir+ strain. This host is a diaminopimelic acid (DAP) auxotroph strain and harbours the *pir* gene to replicate the plasmid R6K origin of replication. After conjugation, pNTM3_*TetA_sacB_KmR* plasmid is integrated into the chromosomal *KmR* copy. Hfr cells, with the integrated plasmid, are selected on LB + tetracycline without DAP. B) Integration efficiency of the pNTM3_*TetA_sacB_KmR* into mutants of the Keio collection is measured as the log10 ratio of CFU per mL of trans-conjugant recombinants (selected on LB + tetracycline) to CFU per mL of donors (selected on LB + DAP + tetracycline). The genes’ names is on the x-axis correspond to the locus in the Keio mutant where the *KmR* gene is integrated. Boxplots represent the results from at least 3 independent replicates (depending on the locus studied, 3 to 26 datapoints were obtained). Boxplots display the median (horizontal line), interquartile range (box) with individual data points shown as dots.

We first tested whether we could obtain efficient and consistent rates of pNTM3_TetA-sacB_KmR plasmid integration into the chromosome via homologous recombination between *KmR* genes by using only the lambda-Red recombinase. We used strains from the Keio collection [25]. Conveniently, this collection is composed of strains, each with *KmR* inserted to replace and inactivate non-essential *E. coli* genes (Fig 2A). We individually transferred the pNTM3_TetA-sacB_KmR plasmid into 14 strains from the Keio collection, as well as a negative control – BW25113 (Parent of the Keio collection, derivative of K-12) lacking *KmR*. In each strain, to allow recombineering, we inserted the plasmid pCREPE [26]. This plasmid contains a chloramphenicol resistance *CmR,* lambda-red recombinase genes as well as an origin of replication pSC101 [27]. After the transfer, we selected for Hfr recipients (Material and Methods), and measured the frequency of integration as the number of CFUs of Hfr variants (presenting *KmR*) relative to the number of CFUs of the pir + pNTM3 donor. As shown in Fig 2B, we observed a low frequency of integration in the negative control. This suggests residual integration independent of the presence of our landing pad. A considerable heterogeneity in plasmid integration rates was observed across genomic loci. Indeed, while we failed to have integration at some positions such as *nfrA*, we obtained integration rates of up to 10^-5^ Hfr CFUs per donor CFU in others positions such as *ybeX* (Fig 2B). The integration rates were, however low overall, below 10^-8^ per donor, and sometimes similar to those of the negative control, suggesting that the Hfr libraries produced will have biased integration sites.

### 2.3. Inducing targeted integration with double-strand break

To improve the integration of pNTM3_TetA-sacB_KmR into the chromosome, we increased the recombination efficiency by using Cas9-mediated targeted DNA double-strand break (DSB) coupled to Lambda-red recombination [28]. Cas9 endonuclease is directed by a guide RNA (gRNA) for targeted DNA double-stranded break (DSB). The gRNA contains a spacer region with a 20 bp homology to the target site, proximal to a 5’-NGG-3’ Protospacer Adjacent Motif or PAM. Cas9:gRNA interaction with the PAM and gRNA::DNA binding at the homologous target site induces the DNA DSB. In *E. coli*, efficient Cas9-mediated targeted DNA DSB is cytotoxic and results in cell death. Cells may acquire immunity to the targeted DNA DSB by replacing the PAM or the spacer sequence by using a repair template and phage-mediated homologous recombination. Therefore, Cas9-mediated DNA DSB can select for precise genome modifications coupled with PAM/spacer mutations introduced using phage-mediated recombination [28]. In addition, recent studies have shown that having a targeted DNA DSB can also improve the efficiency of recombination [29–32]. We induced a DNA DSB in the genomic Kanamycin landing pad using the Cas9. For that purpose, we tested three different gRNAs. The killing efficiency can vary between gRNA and the editing efficiency is correlated to the killing efficiency. Therefore, we first tested the gRNA killing efficiency of the three gRNAs in Keio strains with an integrated copy of the kanamycin resistance gene. All gRNAs had similar efficiencies, so we chose one (sgRNA1) and introduced it into plasmid pNTM3_TetA-sacB_KmR (S1A Fig). To rescue the cells from the targeted DNA DSB using recombination, we modified the corresponding PAM motif in the plasmid-encoded *KmR* gene by introducing a synonymous PAM mutation (SPM). Therefore, genomic integration of the plasmid would replace the chromosomal *KmR* gene with the KmR* gene with the SPM and provide immunity from the Cas9-mediated DSB to select for the plasmid integration. The resulting plasmid was named pNTM3_sgRNA.

**Fig 3:**
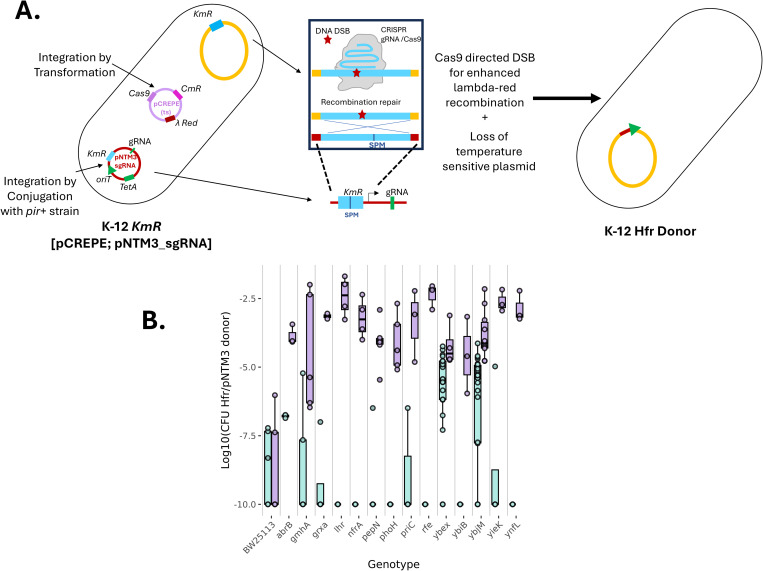
Cas9-Lambda-red recombination-mediated Hfr construction. A) Schematic representation of DSB-induced Lambda-red recombination showing the gRNA and repair template for Cas9-Lambda-red recombination-mediated Hfr construction in the recipient. B) Integration efficiency of the pNTM3_TetA-sacB_KmR into mutants of the Keio collection, measured as the log10 ratio of CFU per mL of recombinants (selected on LB + tetracycline) to CFU per mL of donors (selected on LB + DAP + tetracycline with the Cas9-Lambda-red approach (purple) across the 14 loci and the BW25113 control. For comparison, results obtained without an induced double-strand break (corresponding to Fig 2B) are given in (cyan). Boxplots display the median (horizontal line), interquartile range (box) with individual data points shown as dots for each of the strains.

In each of our Keio strains and the negative control, we transformed the pCREPE plasmid modified to encode Cas9 endonuclease under a constitutive promoter and lambda-red recombination system expressed under a temperature-inducible promoter (at 42^O^C). The pCREPE plasmid has a temperature sensitive origin of replication, due to which it only replicates below 34^O^C (Fig 3A). We induced the system to facilitate recombination between the sequences flanking the cut site in the chromosome and the repair template present on the pNTM3_sgRNA plasmid (see methods) [33].

With the Cas9/recombineering-mediated insertion protocol, we achieved a much higher integration efficiency (S1B Fig). Additionally, we observed less heterogeneity between sites, with an integration rate greater than 10^-5^ for all but one locus (13 out of 14) (Fig 3B). Therefore, Cas9-mediated recombination significantly improved pNTM3 integration efficiency into the chromosome to produce Hfr strains.

### 2.4. Validating the ability of the constructed Hfr to transfer the *galK* marker

**Fig 4:**
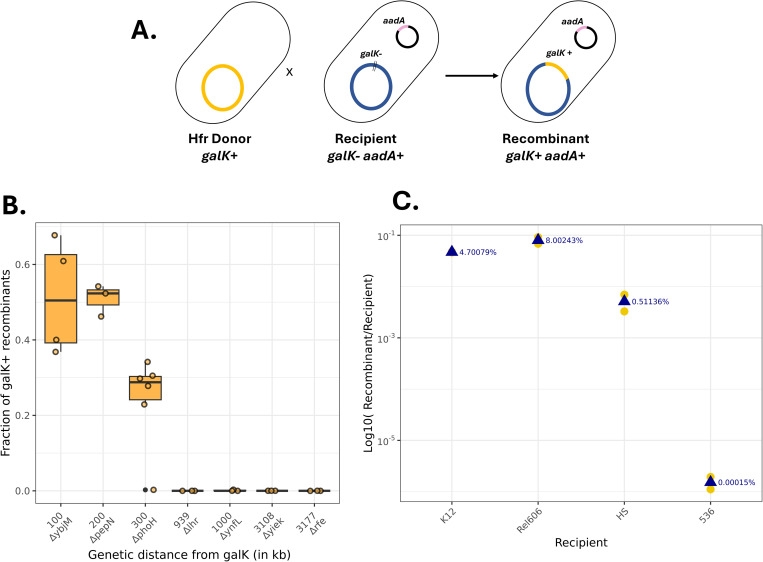
Testing Hfr donor libraries for transfer of a *galK* marker. A) A schematic representing crosses between Hfr *galK*+ donors and *galK*- spectinomycin recipients. Recombinants that have gained a functional *galK* allele are selected on M9 minimal media with galactose and spectinomycin. B) Recombination efficiency measured as the ratio of CFU per mL of recombinant (growing on M9 + galactose + spectinomycin) to CFU per mL of all recipients (selected on M9 + glucose + spectinomycin) for Hfr donors constructed by integration of the conjugative plasmid at different distances from the galK locus. Boxplots display the median (horizontal line), interquartile range (box) with individual data points shown as dots. Statistical comparisons were made using the Wilcoxon test, ns: non-significant, *: p-value ≤ 0.05, **: p-value ≤ 0.01.C) Recombination efficiency measured as the log10 ratio of CFU per mL of recombinant (selected on M9 + galactose + spectinomycin) to CFU per mL of all recipients (selected on M9 + glucose + spectinomycin) for an Hfr donor library crossed with galK- spectinomycin recipients in different genetic backgrounds E. coli K-12 BW25113 (control), REL606, HS, and 536. Means are represented by blue triangles and data points by yellow dots.

To validate that the selected strains were functional Hfr bacteria, we tested their ability to transfer DNA to a recipient strain. To quantify the transfer’s efficiency, we conducted conjugation experiments between Hfr donor strains and a Δ*galK* recipient. The *galK* gene encodes for the Galactokinase protein, which is required for growth on galactose as the sole carbon source. In the Δ*galK* strain, the *galK* gene was inactivated by introducing multiple stop codons had previously been introduced to inactivate the *galK* gene to prevent any possibility of reversion by mutations. Consequently, in this strain, only recombination of a fully functional gene upon conjugative transfer from the Hfr donor could restore the *galK* function (Fig 4A) [26]. In addition, the recipient strain was also rendered resistant to spectinomycin by electroporating a small non conjugative plasmid containing the *aadA* gene. We then crossed each constructed Hfr with the *galK-* recipient and quantified the transfer efficiency as the number of *galK+ specR* transconjugants to the number of *specR* recipient cells. In the Hfr strains constructed using the different Keio mutants, the Hfr-*oriT* was integrated at variable distances from the *galK* gene.

Upon conjugation, we found that the strain bearing the *oriT* inserted at 100kb from *galK* could transfer the *galK+* allele with an extremely high efficiency. Corroborating previous observations, we observed that Hfr-*oriT* integration at a larger distance resulted in lower transfer rates (from 939 kb on, the transfer rates were below detection limit), but the strain with an Hfr-*oriT* at 300 kb could still transfer the *galK*+ allele with 20% efficiency (Fig 4B).

### 2.5. Creating and testing a library Hfr

After functional validation of our targeted Hfr constructs, we tested if we could create a library of Hfr. To construct the library, we used a transposon mutagenesis library in which the *KmR* landing pad is randomly inserted in the chromosome. We constructed the transposon libraries using laboratory strains BW25113 and REL606 [34]. We then used the afore mentioned Cas9/recombineering-mediated system to integrate the pNTM3_sgRNA plasmid across the different sites of integration of the transposon. Upon selection of *TetR* colonies growing on LB plates, we obtained 10^3^ to 10^4^ colonies that had integrated the pNTM3_sgRNA plasmid into the transposon that were pooled in Hfr libraries.

To test if in these Hfr libraries, the integration of pNTM3_sgRNA conjugative plasmid occurred across the genome, we investigated the integration sites. We adapted a method for transposon sequencing to our libraries of Hfr donors to determine the precise locations of the landing pad in which pNTM3_sgRNA was integrated [35] focusing on library made on BW25113. This approach is based on the amplification of a short DNA sequence spanning the extremity of the transposon and the proximal chromosomal sequence (S2A Fig) where the integration has occurred. We then used sequencing to extract the insertion positions. Of note, as the landing pad is larger than 1kb, our approach is indeed identifying the insertion sites of the Kanamycin cassette that were sampled during the creation of the Hfr library. However, our previous experiments have shown the vast majority of integration of the plasmid (from 99 to 99.9%) occurs in the Kanamycin cassette, so the position of the sampled Kanamycin integration sites should be representative of the site of integrations of the plasmids. Overall, these analyses showed that the pNTM3_sgRNA plasmid was integrated at multiple loci across the chromosome.

The genome architecture may lead to biases in the integration of the plasmid. A source of bias is differences in copy number variation between different regions of the genome (with higher copies closer to the origin of replication, compared to the terminus) due to differences between rate of chromosomal replication and cell division. In addition, organization of the *E. coli* chromosome into macrodomains may result in a biased accessibility of their DNA [36]. Additionally, the randomized integration doesn’t guarantee that the distribution of the positions of integration will be homogenous along the genome. For instance, integration inside essential genes will be lethal for the bacteria leading to some local biases of insertion. We therefore investigated the distribution of insertion sites along the chromosome using the genomic coverage derived from transposon sequencing data (the number of reads that match a specific insertion site) (S2B Fig). We compared the density of insertions, calculated on 250-kb bins, along the chromosome as the log2 fold-change in coverage compared to the coverage of the *Ter* macrodomain. There are two ways to analyse the data: we can focus on the insertion sites (red in S2B Fig), or the coverage of these insertion sites (blue on S2B Fig). Insertion sites refer to the identified positions of insertions and likely refer to independent insertions during the process of Hfr construction; we counted 1140 different insertion sites (blue on S2D Fig). Coverage of these sites corresponds to potential differences in the representation of the various Hfr strains in the library, but also integrates potential biases associated with sequencing [37]. As mentioned, higher copy number of the *Ori* macrodomain within the cells usually leads to higher coverage of that region relative to the *Ter* macrodomain in sequencing data.

Analysing density profiles along the genomes with bins of 250kb, we observed that, there was a bias for both metrics: fewer integration sites were found in the *Ter* macrodomain compared to the rest of the genome (S2C Fig) and the coverage of insertions in the *Ter* macrodomain was lower. The bias was visible using both metrics, but was, as expected, more marked considering coverage of insertion sites (up to 10-fold, S2C Fig) than density of insertion sites (up to 3-fold, S2C Fig). This is expected given the afore mentioned *Ori* to *Ter* coverage bias observed in whole genome sequencing. The biases in the density of insertion sites suggest a biological bias during transposon mutagenesis and/or during pNTM3_sgRNA integration, that likely results from the lower number of copies and/or lower accessibility of the *Ter* macrodomain. Such a bias has been reported before, notably by Khetrapal et al [17]. Despite this bias, integrations of the conjugation machinery were spread along the chromosome and our library could therefore be used to transfer any locus in the genome to a recipient strain.

To test if this library could transfer DNA to various genetic backgrounds, we mated the donor K12 BW25113 Hfr library (constructed twice independently) with 4 recipients with different genetic backgrounds: the laboratory strains BW25113 and REL606, the natural isolate HS [38], and the pathogenic strain 536 [39]. While the first three correspond to strains from phylogroup A of *E. coli*, the latter from B2 phylogroup is more genetically distant. All four strains were made *galK-* and resistant to spectinomycin via the presence of a plasmid. After the mating with the Hfr library, we plated on minimal media with galactose and spectinomycin to select only for transconjugants that recovered a *galK+* allele and get rid of donors and recipients. We estimated the recombination efficiency as the ratio of number of recombinant colonies (selected by plating minimal media with galactose and spectinomycin) to the number of recipient colonies (estimated by plating on non-selective minimal media with glucose and spectinomycin). The frequency of recombinants varied largely depending on the recipient’s genetic background (Fig 4C). In BW25113 and REL606 backgrounds, the recombination efficiency was up to 5–10%, This efficiency reduced to ~0.5% in HS giving only a few colonies of recombinants and it goes as low as 0.00015% when 536 was the recipient. This difference is correlated with the genomic divergence between K12 and the recipients. Additionally, this effect might be amplified for the strain 536 compared to HS or REL606, as we found that 536 is expressing toxins that might kill the donor strains during the conjugation assay (S3 Fig) and harbours also a candidate restriction system of type 2 that could degrade the incoming DNA [40].

### 2.6. Analysis of the genome of the recombinants

**Fig 5:**
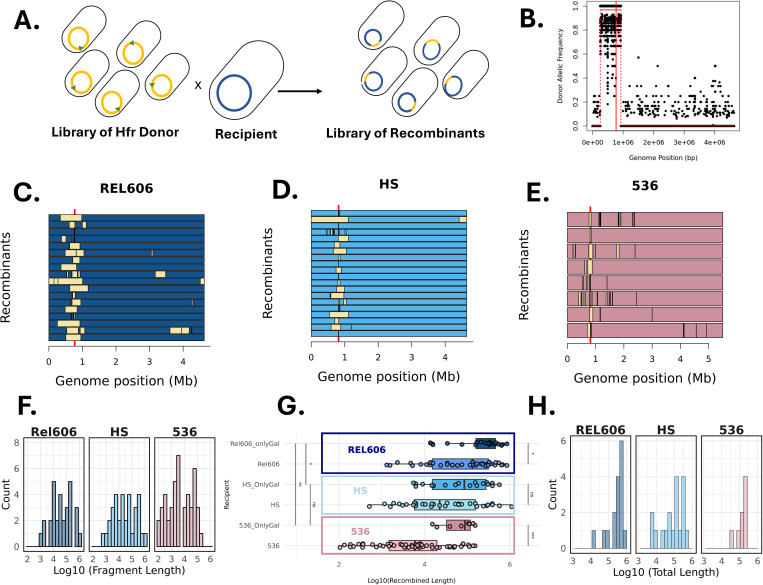
Genome sequencing of recombinant clones. A) A schematic representing the creation of recombinants from a conjugation between an Hfr library used as donor and a recipient. B) Identification of the recombined region of a recombinant. Frequency of donor alleles along the genome of a REL606 recombinant. The red line shows the *galK* locus position. The broken line shows the delineation of the recombined fragment using the changepoint package. D, E) Recombined fragments (yellow fraction) across the genomes for recipient REL606 (C), HS (D) and 536 (E). Red line indicates the *galK* loci position. F) Distribution of recombination fragment length (log10 scale), colours correspond to the different recipients (REL606, dark blue, HS, light blue, 536 light pink). y-axis represents the count of recombinant genomes, the bins height corresponds to the number of a recombined fragment in a certain length interval. G) Boxplot showing the size of recombination fragments for each recipient (log10 scale), for fragments including the selected marker (upper box) and the ones not including it (lower box, lighter colour). Wilcoxon-test was used for statistical analysis. ns: non-significant, *: p value ≤ 0.05, **: p value ≤ 0.01. H) Distribution of the total length recombined (log10) per genome of recombinants (REL606, dark blue, HS, light blue, 536 light pink).

Since we initiated the transfers from multiple loci, we expected to observe different patterns of recombination along the genome (Fig 5A). For the various crosses we made, we sampled 19–20 clones for sequencing (only 8 in the case of 536 due to low efficiency) and determined the parts of their genomes that came from the donor or the recipient strain.

To identify the recombined tracks, we used two independent methods that produced almost identical results. In the first approach, we focused on read mapping. Sequencing output, consisting of 150-bp reads, was mapped competitively against the donor and recipient genomes. Three outcomes were possible. (i) Reads could match both genomes equally well, in which case they were not informative. (ii) Reads could match only one of the two genomes, thereby providing information on accessory genes that were retained or transferred. Since our interest here is in the consequences of recombination on the core genome, we did not use these reads. (iii) Reads could match both genomes but with a preference for one over the other, owing to the presence of alleles specific to one of the strains. The positions of reads matching preferentially to the donor genome were then used to delineate genomic fragments that originated from the donor in a given recombinant. To do this, we applied a Hidden Markov Model to define transitions between two states: donor-derived and recipient-derived. This method is relatively robust to sequencing errors because it considers the entire read for mapping, and in most cases a 150-bp stretch of the core genome contains more than one difference between the donor and recipient genomes.

As an alternative approach, we relied on allele frequencies at polymorphic sites between the donor and recipient core genomes. We first identified all polymorphic sites and then mapped reads to compute the frequency of donor and recipient alleles along the genome. A step function was then used to detect transitions between regions enriched for donor or recipient alleles. This method offers the highest possible resolution but can be more sensitive to sequencing errors. Comparison of the two methods yielded nearly identical (S4 Fig) and robust detection (S5 Fig) of recombined fragment and estimation of fragment length and revealed as shown in Fig 5B–E, that all genomes but one exhibited a clear recombined region around the selected site.

The patterns of recombination were however quite different between recombinants and between recipient strains (Fig 5C, 5D and 5E). First, 22 recombinants (1/8 for 536, 7/18 REL606 or 14/20 for HS) showed a single fragment of recombination that overlapped the *galK* but the length of this fragment was very variable: from a few kilobases (1.5 kb or 7.5 kb), up to a full megabase with a fragment as long as 1.103 Mb. Transfer size can therefore span about 3 orders of magnitude. Second, the 23 other recombined strains harboured not only a recombined fragment around the locus of selection *galK*, but also several other recombined regions in the neighbourhood. They contained between 2 and 16 fragments including the one containing *galK*. This is compatible with an incoming DNA fragment being cut and recombined into pieces [6]. This pattern was most drastic with strain 536 as recipient with 7.125 fragments per recombinants compared to 1.65 for HS and 2.22 for REL606. We also noticed that a few recombinants revealed some distant distinct sites of recombination (5/19 for REL, and 3/8 in 536). Finally, a few strains had not resolved the recombination in the colony that was sequenced as the recombined regions appeared to have both overlapping donor and recipient-specific sequences.

Overall, the distribution of the recombined fragments varied across strains (Fig 5C–G). The median size of recombined fragments was 198,195 bp, 126,214 bp and 21,353 bp for the recipients REL606, HS and 536 respectively (Fig 5F and 5G); when considering only fragments that included the *galK* locus, their lengths were significantly greater across all strains (327,529, 156,831.8 and 82,130 bp median size in REL606, HS and 536 respectively) (Fig 5G).

We tested the lengths’ differences between the *galK-*containing fragments across recipient strains and found that there was a significant difference between REL606 recombinants compared to HS and 536 recombinants (Fig 5G). When we looked at the total length of recombined material, the difference between strains remained with the mean sizes being 152kb, 208kb and 440kb for 536, HS and REL606 respectively (Fig 5H).

### 2.7. Expected power to detect a selected site

Sequencing of individual clones confirmed the occurrence of recombination and provided critical insights into the length, position, and number of recombined fragments. Beyond this descriptive analysis, the experiment can be viewed as a genetic cross coupled with selection, which enables the detection of loci under selection in a QTL-like framework. However, unlike the genetic exchanges typical of eukaryotic systems used in QTL approaches, the recombined fragment lengths here are highly variable. This raises the question of how such variability impacts the ability to pinpoint loci under selection.

To explore this generically, we employed mathematical modelling. We analyzed the statistical properties of recombined fragments conserved among a pool of selected recombinants near the allele under selection. The genomic region where donor DNA is consistently found across all selected recombinants is likely to contain the selected allele—here, the functional *galK* allele. Recombined fragments were modelled as independent, identically distributed intervals with positions uniformly distributed across the genome. This model demonstrated that for a large number (n) of selected recombinants, the length of donor DNA shared by all recombinants scales with the harmonic mean of the length of the recombined fragment encompassing the selected loci divided by the number of recombinants. Specifically, the expected size of the region likely to harbour the selected locus, denoted as, $\Lambda_{n}$, is given by

$\text{E}\left(\Lambda_{n}\right)=\frac{\text{2}\mu }{n\;}\;$, where $\mu =\frac{\text{1}}{E\left(\frac{\text{1}}{L}\right)}$ represents the harmonic mean of the recombined fragment lengths that include the selected loci (see Proposition 4.1 in S1 File).

This result is significant because the harmonic mean is particularly sensitive to smaller values, meaning that the presence of short fragments—just a few kilobases in length—can substantially reduce the size of the shared recombined region across recombinants. Consequently, the model suggests that the observed heterogeneity in recombined fragment lengths may, counterintuitively, enhance the precision of locus detection under selection.

### 2.8. Analysis of the pool of recombinants

To test our ability to detect with precision the selected loci, we decided to use another approach based on sequencing a pool of recombinants. Using this methodology, we can analyse the whole population and look at the density of donor-specific reads along the genome to visualize patterns of recombination at the pool scale.

**Fig 6:**
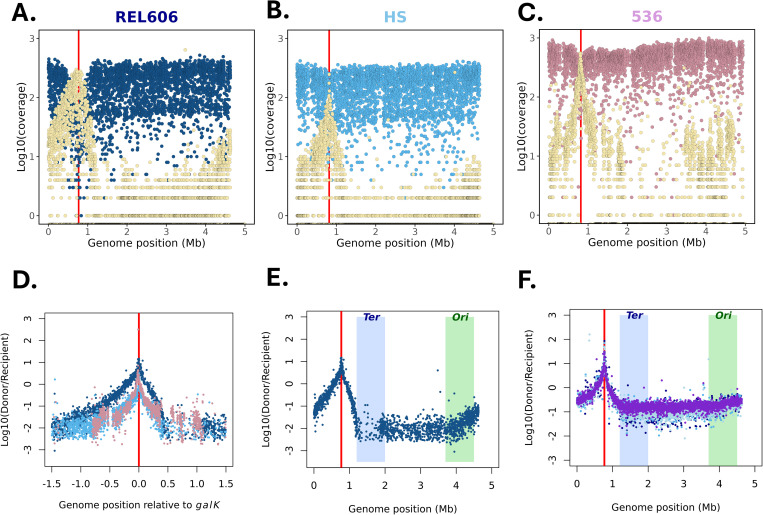
Pooled Genome sequencing. A, B, C) Log10 of the per kb coverage of the genome with reads preferentially mapping to the recipient genome (in A dark blue for REL606, in B blue for HS, in C pink for 536) or the donor one (yellow). Red lines show the *galK* loci position. D) Log10-ratio of donor-to-recipient coverage along the genome relative to *galK* position for the three recipient genotypes (dark blue for REL606, blue for HS, red for 536.E) Log10-ratio of donor-to-recipient coverage along the genome for REL606 pool with the Terminus *Ter* (light blue) and *Ori* (green) macrodomain positions shown. F) Log10-ratio of donor-to-recipient coverage along the genome for three different ∆*matP* REL606 mutans (purple, light blue and median blue) and REL606 (dark blue). The red line shows the *galK* loci position with the Terminus *Ter* (light blue) and *Ori* (green) macrodomain positions represented by colored rectangles.

Because we are enforcing a strong selection at *galK* loci, we expected a clear and strong signal of donor DNA integrated into the recipient. For each of the recipients’ backgrounds, we identified a peak of donor-specific sequences around the *galK* loci (Fig 6A, 6B and 6C). The delineation of the target region was even more precise using the ratio of donor to recipient-specific reads along bins of 1 kb along the genome. The maximum of that ratio landed exactly in the *galK* gene for all crosses (Fig 6A, 6B and 6C). The pattern of decay of the donor DNA presence differed between the recipient strains (Fig 6D) with a decay less marked in REL606 than in HS and 536. This pattern is consistent with the distribution of recombination length detected as longer recombination tracks should lead to a slower decay.

For all recipients, we observed a steeper reduction of donor DNA towards the *Ter* macrodomain (Fig 6E). This pattern could result from the integration bias of the conjugative plasmid in our library. However, we also know that the *Ter* macrodomain is structured and made less accessible through the interaction between the MatP protein and the *matS* sites that are frequent in that macrodomain [36]. To test if that structuration of the *Ter* Macrodomain could reduce the efficiency of recombination and contribute to the observed pattern, we used a ∆*matP* mutant as a recipient. We observed no difference between ∆*matP* and the wild type (Fig 6F) suggesting that recombination was not affected by macrodomain structural organization and that the biases resulted most likely from our skewed distribution of conjugative plasmid integration sites.

### 2.9. Selection at different loci and precision of the genomic analysis

We demonstrated through the *galK* crosses that we could extract critical insights into the recombination patterns by using pools and into the variability of recombined regions by using individual clones. To verify that these observations were not artefacts stemming from the chosen selection locus or its chromosomal position, we generated a new library of REL606 recipients using an alternative marker for counter-selection. By employing transposon mutagenesis, we randomly inserted a cassette containing the toxin *tse2* under the control of a rhamnose-inducible promoter [41]. Upon rhamnose induction, these mutants expressed the toxin and undergo cell death, enabling the selection of recombinants that have replaced the *tse2* gene thanks to the incorporation of donor DNA, as the donor lacks the toxin. From this library, we selected three individual clones as recipient with *tse2* cassette inserted at distinct random positions. To counter-select against donor strain during the crosses, we also integrated a resistance gene for zeocin at a fixed position into the chromosome of the recipients.

**Fig 7:**
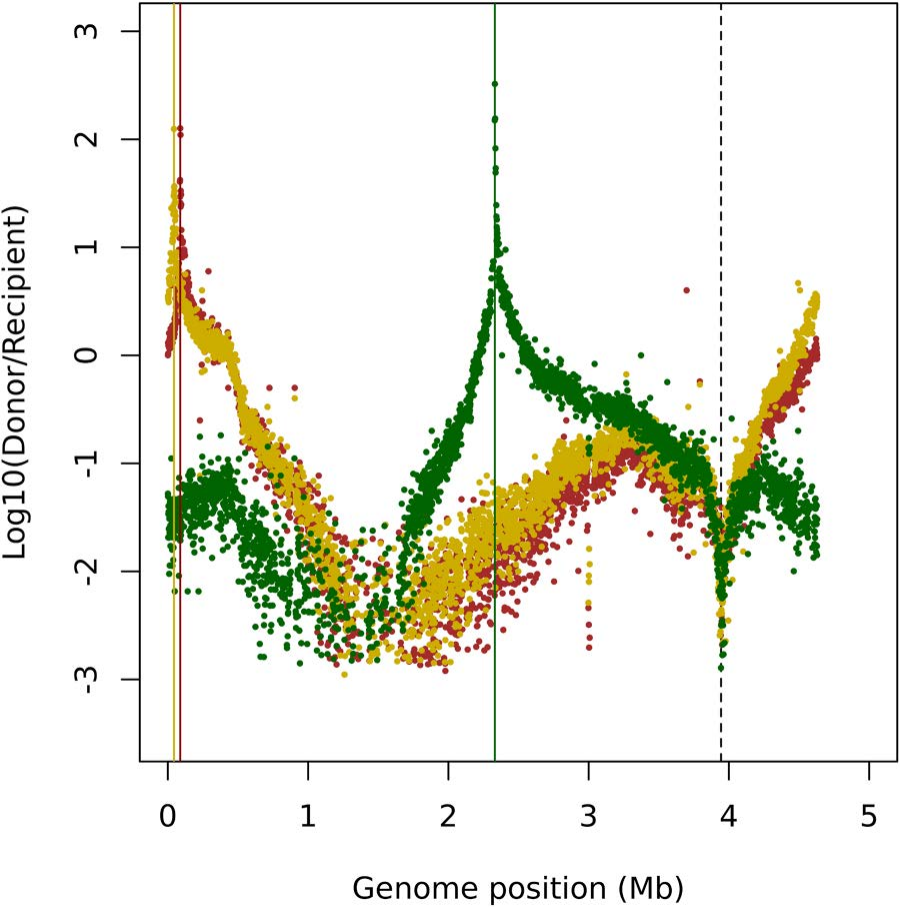
Pooled Genome sequencing of *tse2* recombinants. Log10-ratio of donor-to-recipient coverage along the genome for the three REL606 recipients with different *tse2* positions (respectively in dark red, yellow, dark green). The vertical lines (red, yellow, dark green) correspond to the maximum of the log10 ratio Donor/Recipient for each of the sequenced pools (*tse2* position in the recipient). The black dotted line corresponds to the position of *ZeoR* present the recipient background and used for selection.

We then recombined these recipients with a library of *E. coli* K-12 BW25113 donors. Genomic analysis of the resulting recombinant pool provided profiles of recombination events (Fig 7). By analyzing the ratio of donor- to recipient-specific reads, we observed that the selection pattern closely resembled the one seen with the *galK* crosses. Selection effects were highly localized and specific across all pools. In addition, since recipients and transconjugants were now selected using the chromosomal *zeoR* marker, a sharp decrease in the ratio donor-to-recipient coverage was observed at the z*eoR* insertion site.

The next question was whether we could localize the position of *tse2* insertion sites with the same precision as the one achieved with the *galK* locus, where three independent replicates yielded an accuracy of 1 to 1.5 kilobases. To address this, we analyzed the chromosomal position of the maxima in the log10 ratio of Donor/Recipient reads (Fig 7) for each pool of recombinant. This peak serves as our best estimate of the location of the *tse2* cassette in the recipient strain. We then designed primers positioned 1 kb and 1.5 kb upstream and downstream of the inferred peak, along with primers specific to the *tse2* cassette itself (S6 and S7 Figs). PCR amplification using the 1 kb primer set successfully detected the cassette in the inferred region for two out of the three samples. For the third sample, detection required pairing primers 1.5 kb from the inferred peak with those targeting the cassette (S7 Fig). Thus, across all three samples, we identified the position of the locus under selection with an accuracy of ±1.5 kb. Therefore, we showed that our method could be used for efficient and precise QTL mapping.

## 3. Discussion

In this study, we present an alternative high-throughput approach for genomic conjugation. This method relies on the generation of a library of mutants called Hfr donor libraries, each mutant having a different chromosomal integration site for a conjugative plasmid. To promote random integration into the chromosome, we employed homologous recombination between the plasmid and a transposon cassette, the latter having been randomly integrated into the genome via transposon mutagenesis. To enhance integration rates and reduce biases, we resorted to CRISPR-Cas9 system to induce double-strand breaks in the chromosome, alongside the use of lambda Red recombination machinery to enhance recombination efficiency.

Before discussing our results, it is important to acknowledge several limitations of our protocol. One major limitation of our study lies in the use of a specific conjugation system while many different conjugative elements exist, previous relied on plasmids RP4 [17], we used here an F derivative, F plasmids being the most common conjugative plasmids in *E. coli* [42]. Second, in the present study, we focussed so far on the selection at a specific marker though we showed the patterns were conserved at other sites. Another issue is that our library of donors is not homogeneously spread around the chromosome presumably due to reduced integration of the transposon and integration of plasmids in the *Ter* macrodomain near the terminus of replication as previously observed [17]. This prevents us from using our protocol to investigate the heterogeneity of recombination along the chromosome observed using comparative genomics [43]. Despite these limitations, our protocol allowed us to successfully generate recombinant libraries from crosses between the K-12 laboratory strain and three different *E. coli* strains.

Genomic analysis of these recombinant libraries revealed an unexpected diversity in the lengths of donor fragments incorporated, ranging from a mere kilobase to over a million base pairs. Earlier genome-wide studies had suggested that recombined fragments were typically in the tens of kilobases, and therefore associated with bacteriophage-mediated transduction [9,10], while conjugation was linked to larger transfers involving several hundreds of kilobases. Our findings indicate that, even in tightly controlled laboratory settings, conjugation can yield extensive diversity in recombined fragments. This suggests that even short recombined fragments detected in genomic studies could originate from a conjugation event.

Several recent approaches have been developed to perform MAE. Although these studies did not explicitly focus on fragment-length variation, they nonetheless reported substantial heterogeneity in the size of recombined DNA segments. In *E. coli*, Khetrapal et al. [17] observed transferred fragments ranging from 16kb to 1.78 Mb. The plasmid employed in their work differed in type from that used in the present study, suggesting that variation in fragment length can arise independently of plasmid backbone.

This observed variability in recombined fragment size has significant implications for the precise identification of loci under selection. By analysing the length variability and distribution of fragments, we can pinpoint genomic regions subjected to selection with 1–2 kilobases precision. Mathematical modelling suggests that when selecting for a specific allele from a donor strain, the size of shared fragments among recombinants correlates with the harmonic mean of the length of the recombined fragment encompassing the selected loci. Since the harmonic mean is sensitive to smaller values, the broad size range we observe enables precise identification of selected loci. This leads to two key consequences: first, it allows for the identification of causative alleles under strong selection; second, it suggests that conjugation may influence natural genome evolution in unforeseen ways.

Detecting the genetic source of a strain specificity remains an important challenge in microbiology. Within the same species, a large variability of phenotypes is observed including some traits of importance such as virulence or antibiotic resistance [44,45]. For many of these traits, gene presence and absence are the major sources of diversity, and alternative methods including transposons mutagenesis have been used for decades with success. However, some important phenotypic diversity is explained by allelic variation within conserved regions of the genome rather than gene presence and absence as seen in experimental evolution [12,46]. To discover these types of variations MAE as described here or in Khetrapal *et al.* [17] may be relevant, especially using the low-cost pooled sequencing that we have used. However, further investigation will be needed to test the efficiency of MAE when the locus studied is under moderate selection.

The considerable diversity in recombined segments challenges also the existing assumptions about genetic linkage and bacterial conjugation. While conjugation was supposed to involve large fragments, our results suggest that the size of the block transferred in full linkage is much lower than expected. This finding introduces an element of unexpected plasticity in bacterial genome evolution relying on conjugation. The sharp decline in the fraction of donor DNA around selected loci indicates that following transfer and subsequent selection, genetic diversity within the population is largely preserved. This low linkage may account for the mosaic nature of bacterial genomes and their rapid adaptability to diverse environments.

This plasticity in the size of recombined fragments is reminiscent of what has been reported for natural transformation in other species. Initially, transfers were thought to involve only short fragments, typically on the order of a kilobase [47,48]. It is only more recently that transfers of 100 kb or more have been documented, notably in *Vibrio cholerae* [49]. Another mechanism, distributive conjugal transfer, described in mycobacteria, also produces fragments of variable size, but here the number of fragments exchanged in a single mating event is much higher [50]. In all these cases, flexibility in the size of genetic material exchanged provides a spectrum of opportunities for adaptation. Small fragments can break linkage between closely related strains, whereas very large fragments allow the co-transfer of functional gene clusters, such as entire operons or genomic islands.

Our analysis also identified variations in recombination profiles among recipient strains, hinting at an inverse relationship between recombined fragment sizes and the phylogenetic distance between donor and recipient strains. Several factors could account for this variability. First, defence mechanisms, such as restriction-modification systems, might fragment incoming DNA, leading to lethal consequences for bacteriophages but allowing multiple integrations of homologous DNA [6]. This was indeed observed for natural transformation [47,48,51]. Strain 536 which showed the highest number of recombined fragments harbours for instance a candidate type two restriction system according to predictions [40]. Additionally, sequence insertions and sequence divergence may reduce the number of perfect homology sites necessary for recombination initiation, thereby hindering integration (38). Nevertheless, because the strains share a large fraction of their genes with high sequence similarity, potential sites for recombination initiation are abundant across the chromosome (S8 and S9 Figs). This suggests that limited initiation opportunities are unlikely to be the main factor driving the phenomenon. Finally, selection may disfavour strains harbouring recombined fragments due to their disruptive effects on genomic organization. All these hypotheses have to be further investigated notably by applying our protocol and others to a large number of strains and selected mutants.

## 4. Materials and methods

### 4.1. Growth conditions

Liquid cultures were performed in LB Miller or M9 minimal media supplemented with glucose 0.4% or galactose 0.4%. LB was purchased from Life Technologies (Thermofischer) (reference 12795027) with 5 g/L yeast extract, 10 g/L tryptone, and 10 g/L NaCl; 15 g/L agar added as needed. In each 100 mL M9 media, we added M9 salts (prepared using premixed powder from BD Biosciences Difco 5X (reference 248510)), 0.4% Glucose or Galactose or Rhamnose, 2 µM MgS0_4_, 0.1 µM CaCl_2_, and Thiamine 0.2 µg/mL. For solid cultures in Petri dishes, 1.6% Select agar was added. Recombination efficiencies were measured using Bacto McConkey agar with 0.4% galactose [15]. When appropriate, antibiotics were added to the medium as follows: chloramphenicol (34 µg/ml), kanamycin (50 µg/ml), spectinomycin (50 µg/ml), gentamicin (15 µg/ml), tetracycline (10 µg/ml) or zeocin (50 µg/ml). For negative selection on *tse2* cassette, 0.2% rhamnose was added to the M9 minimal media.

### 4.2. Strains, plasmids and cloning

A complete list of the strains, plasmids and primers used in this study can be found in Supplementary material (S1–S3 Tables) s. To construct libraries of Hfr donors, we initially used the suicide conjugative plasmid pNTM3, a derivative of the F-plasmid engineered to have a *pir-* dependent R6K origin of replication and a spectinomycin cassette for selection (11). This plasmid replicates in a *pir +* *recA-* host (*ΔdapA::pir;* strain BW38029) from which it can be transferred by conjugation. To facilitate further selection, pNTM3 plasmid was modified by lambda-red recombineering using the Datsenko-Wanner method (43) to introduce a cassette encoding a resistance gene to tetracycline (*TetA*) and a gene conferring sucrose sensitivity (*sacB)* (S1A Fig). This cassette was amplified from strain XTL634 [52]. The resulting pNTM3 serves as a conditional integration vector: maintained and replicated in *ΔdapA::pir* strains whereas upon transfer to a *pir- recA + F-* strain, it will be integrated in the chromosome by homologous recombination.

Secondly, a *KmR* gene, amplified from the plasmid pKD4, was inserted into pNTM3 using the same lambda red strategy, generating the plasmid pNTM3*_TetA_sacB_KmR.* This construct was initially used to test chromosomal integration efficiency using clones from the Keio collection (Fig 2A). As we obtained low levels of chromosomic integration by using this pNTM3*_TetA_sacB_KmR* construct, the plasmid was further modified to incorporate a gRNA targeting *KmR.* The corresponding PAM motif present in *KmR* was mutated to a synonymous codon to prevent re-cutting by Cas9 post recombination (S1A Fig). To this end, an intermediate vector, pVec, was constructed by Gibson assembly using 1) the backbone of a plasmid containing the *colE1* origin with the *aadA* gene and 2) a fragment of the pNTM3_*TetA-sacB_KmR* encompassing *finO, KmR, TetA, sacB* and the R6K origin of replication. The PAM motif present in *KmR* was mutated in pVec by using the Q5 Site-Directed Mutagenesis kit (New England Biolabs) and oligos kan_SDM_R and kan_SDM_F. The sequence of gRNA1 under the J23119 promoter (see below) was inserted in place of *sacB* by using Gibson assembly and primers TC_gRNA_INS_F and TC_gRNA_INS_R.

To test this CRISPR-cas9-recombineering-mediated Hfr construction, we constructed individual donors using the strain *E. coli* K12 BW25113 (*lacI^q^ rrnB_T14_ ΔlacZ_WJ16_ hsdR514 ΔaraBAD_AH33_ ΔrhaBAD_LD78_*) and its derivatives from the Keio collection where several genes were disrupted with a kanamycin resistance gene: *abrB, gmhA, lhr, nfrA, pepN, phoH, priC, rfe, ybeX, ybiB, ybjM, yieK, ynfL.* For CRISPR cas9-recombineering-mediated genome modification, we used pAM053 plasmid [53] encoding a constitutively expressed *cas9,* heat-inducible lambda red recombination proteins, chloramphenicol resistance as well as a temperature sensitive origin of replication. Mutations in *galK* were introduced by CRISPR-cas9-mediated recombineering using a construct named G22 previously reported [15] and a gRNA1 expressed under the J23119 promoter using the gRNA plasmid purchased from Addgene (https://www.addgene.org/71656/). Similarly, for testing the gRNA efficiency to direct DSB into KmR, three gRNAs (sequences in S4 Table) were cloned into the gRNA plasmid by using the primers kan_gRNA1_f, kan_gRNA1_r, kan_gRNA2_f, kan_gRNA2_r, kan_gRNA3_f and kan_gRNA3_r. The *KmR* cassette for transposon mutagenesis was amplified using the pSAM_*Kan* plasmid.

In the recipient strains, a Zeocin resistance gene was integrated into the chromosome at Safe site 9 [54] by using plasmid pSS9 (https://www.addgene.org/71655/).

### 4.3. Lambda-red recombination and CRISPR-cas9-mediated recombineering

The protocol for CRISPR-Cas9-recombineering and Lambda-red recombineering were the same. We started overnight cultures of cells in LB supplemented with Chloramphenicol containing either a plasmid encoding *lambda-red* recombination genes or a plasmid containing *cas9* and *lambda-red* recombination genes at 30°C. The next day, fresh 100 mL cultures were inoculated with a 100-fold dilution of the overnight cultures in fresh media and grown at 30°C up to a mid-log OD measured at 600 nm (OD600) of 0.4–0.5. The flasks were then placed in a shaking water bath set at 42°C for 15 minutes to induce the *lambda-red* recombination operon. After 15 minutes, the cells were immediately placed on ice and chilled for 15–20 minutes. The cells were then centrifuged at 7,500 × g at 4°C for 3 minutes. The media was discarded and the pellet was resuspended in 25 mL ice-cold 10% glycerol and resuspended gently. The pellet washing step was repeated with 25 mL 10% glycerol 5 times and with 10 mL 10% glycerol one final time. The pellet was finally resuspended to a final volume of 300 µL in 10% glycerol. 100 µl of competent cells were mixed with at least 100 ng of DNA or plasmid, previously dialyzed on a 0,025 µm membrane. The cell-DNA mixture was electroporated at 1.8 kV in 1mm electroporation cuvettes and recovered using 1 mL of LB media in 1.5mL tubes. The cells were recovered for 2–3 hours at 30°C and then plated on LB agar plates with the required antibiotics.

The protocol for making competent cells for electroporation of other plasmids was the same as above but with two differences: 1) There was no induction step. 2) The cells were grown at 37^°^C unless they harboured a temperature-sensitive plasmid.

### 4.4. Construction of the transposon library

The transposon library was prepared either using EZ-Tn5 transposase or by conjugation between recombination-proficient MDF *pir*+ cells with the plasmid pSAM_*Kan* containing a Mariner C9 transposase (following the conjugation protocol below). When using the hyperactive form of Tn5, we constructed our transposon library by mixing EZ-Tn5 transposase (BioSearch technologies, Kit TNP92110) with a *KmR* cassette from the plasmid pSAM_*Kan.* The cassette underwent the following steps before the transposon reaction. The cassette was first amplified by PCR with the primers Ez_Tn5_Kan_for and Ez_Tn5_Kan_rev to add the ME transposase recognition sequence. The PCR product was purified on gel, extracted using gel purification and dialyzed afterwards for 1h using a 0.025µM dialysis membrane to remove excess salts. Subsequently, DNA was phosphorylated to increase its stability *in vivo* using the NEB T4 polynucleotide kinase (PNK) enzyme mixed as 44µL of DNA + 1µL of T4 PNK + 5µL of T4 DNA ligase buffer with ATP. The reaction was incubated at 37^°^C for 60 minutes. This final construct was then mixed with the Ez-Tn5 transposase as: 1 µL of PCR product + 1 µL of 100% glycerol + 1 µL of ultrapure DNase free RNase free water + 1 µL of Ez-Tn5 transposase. This reaction was incubated for 60 minutes at 23^°^C. Subsequently, the DNA-transposase mixture (transposome) was dialyzed for 30 minutes to 60 minutes using 0.025 µm dialysis membranes. 0.5 to 1 µL of the dialyzed product was electroporated into the desired competent cells to make the transposon mutagenesis library. The product could also be stored at -20^°^C.

### 4.5. Conjugation

Conjugation experiments were performed to fulfil three different objectives: (i) Construction of a library of KmR-Mariner transposon mutagenesis library, (ii) Generating Hfr donors: integration of the F-factor plasmid into the donor chromosome. (iii) Producing of recombinants from Hfr strains or libraries. For all these conjugation experiments we relied on a protocol adapted from Ma et al. 2014 [55].

Overnight cultures were started for the donor and the recipient strains in 5 mL LB with their respective antibiotics to select for the selection markers and plasmids. If the recipient was a library, we started 100 mL overnight culture in LB using 500 µL of the library glycerol stock. When using *pir + dapA-* [pNTM3_TetA_sacB_KmR] strain as a donor, the media was supplemented with 0.3 mM diaminopimelic acid (DAP) as this strain is auxotroph for DAP. On the subsequent day, fresh 50 mL cultures were launched for the donors and recipients in LB and their respective antibiotics for selection. When the donor or recipient contained a temperature-sensitive plasmid, the cells were grown at 30^°^C. When the donors and recipients reached an OD of 0.5-0.6, they were centrifuged at 8,600 g for 3 minutes. When constructing donor Hfr, the cells were induced at 42°C after they reached the OD of 0.5-0.6 and placed on ice right after.

For all purposes, the cells were washed with 10 mL physiological water and centrifuged at 8,600 g for 3 minutes at 4^°^C. The washing step was repeated twice. The cells were finally resuspended in 1 mL of physiological water. Subsequently, the OD600 was measured on diluted samples: 10 µL of culture in 990 µL of fresh medium in a spectrophotometer cuvette and measured on a spectrophotometer. The samples were the normalized according to their OD600 to combine donors and recipients in a 4:1 ratio by mixing 80 µL + 20 µL of cells. The combined cells were thoroughly mixed and pipetted onto a prewarmed agar plate without antibiotics as two 20-µL spots and six 10-µL spots. All agar plates were transferred to 37°C for overnight incubation for Lambda-red/cas9 mediated Hfr donor construction or for 3 hours at 37°C for recombinant production. The cells were removed from each plate by rinsing 750 µl of fresh medium over the plate multiple times and centrifuged at 13,500g for 1 min at room temperature. They were then serially diluted up to 10^−4^ and the dilutions were plated on LB agar square plates with the required selection antibiotics.

### 4.6. Quantitative determination of anti-bacterial activity assay

To measure the antibacterial activity of candidate attacker *E. coli* strains against the target strain K12::*ZeoR,* we used an adapted version of Alcoforado Diniz et al. protocol [56]. We used K12 without Zeocin resistance mixed with K12::*ZeoR* as a negative control. IAI14 and IAI35 mixed with K12::*ZeoR* are positive controls (known toxin producing strains) from IAI collection [57]. Screened strains HS and 536 which cannot grow on zeocin were mixed with K12::*ZeoR.* Biological replicates were performed. Strains were grown overnight at 37°C in LB agar plates. The next day, plates were scraped, and cultures were resuspended in 0.5mL sterile LB. OD600 was measured and normalized to 0.5 for a volume of 100µL. After OD normalization, the strains were mixed at a 5:1 ratio (potential killer vs K12::*ZeoR*) plated as 25µL spots on fresh LB agar plate and incubated at 37°C for 4 hours. Each spot was then scrape and resuspended in 1 mL LB. Plating was done on LBA Zeocin, by serial dilution to quantitatively measure bacterial survival from undiluted to 10–6. 5µL spots on LB + Zeocin plate were performed and incubated at 37°C overnight. Next day, the colonies were counted.

### 4.7. Statistics and reproducibility

All statistical analyses were performed in R (R Core Team, 2022).

Tests used are indicated in the text or figures’ legend. All data are representative of at least two biological replicates. When two groups were being compared and more than 5 data points were provided, a Wilcoxon test was used. p-values below 0.05 were considered significant.

### 4.8. Genome sequencing

WGS was performed using Illumina technology. DNA samples were extracted using the Genomic DNA NucleoMag 96 tissue kit from Macherey-Nagel. The whole-genome sequencing libraries were prepared and indexed using the Illumina DNA Prep Tagmentation kit and IDT for Illumina DNA/RNA UD Indexes kit A/B/C/D. The paired-end sequencing was performed with Illumina MiniSeq or NextSeq 500/550 to a read length of 2 x 150 bp. The genomes were sequenced at an average depth of 100× for the pooled libraries and 10X for individual clones. Illumina read sequences were deposited at NCBI (Bioproject PRJNA1219123).

### 4.9. Bioinformatics

For strain 536, we used the sequence of the isolate maintained in our laboratory [58].

Two approaches were used to identify the recombined fragments within the genomes of the recombinants. In the first approach, we used the aligner bwa [59] to competitively map all reads against the donor and recipient genomes. The mapping outcome was then used to categorize reads into different groups. Since each read is associated with a genomic position, these categories can be used to infer which part of the recombinant genome originates from the donor or the recipient. Some reads matched both genomes with identical scores; these are uninformative, as they do not reveal whether the aligned region derives from the donor or recipient. Other reads matched only one genome but not the other; these correspond to regions specific to one strain that are present in the recombinant. Because we focused on the core genome—*i.e.,* the regions conserved between both genomes—such reads were not used. Finally, some reads aligned to both genomes but with different alignment scores; these are informative, as they indicate whether the corresponding genomic region derives from donor or recipient origin. For each such read, we recorded its position in the recipient genome and categorized it as 1 if it matched better to the donor genome, and 0 otherwise. Once ordered by genomic position, we applied a Hidden Markov Model to assign contiguous genomic regions to either donor or recipient origin. This analysis was performed using the R package HMM.

For pooled sequencing data, we used the same approach but without the Hidden Markov Model step. Instead, we computed for each 1 kb genomic window the fraction of reads categorized as 1.

To further validate this approach and obtain potentially better-resolved recombination junctions, we developed an alternative method based on allele frequencies along the genome. The principle here is that, for all core-genome sites that differentiate the two strains, we compute the frequency of alleles corresponding to the donor genome.

Polymorphic sites within the core genome were first identified using Parsnp 2.0 [60]. The genomes of both donor and recipient were provided as input, with the recipient genome set as the reference. Parsnp outputs a multiple alignment in.xmfa format, containing all homologous sequence blocks between the two genomes according to their positions in the reference genome. To refine these alignments, we applied a filtering step using a Perl script to retain only syntenic fragments shared between the genomes. Blocks shorter than 110 bp were excluded, as these often corresponded to spurious homologies in accessory genes not in synteny. We also discarded blocks located more than 100 kb away from the previous block, or those located on the opposite strand, as these correspond to synteny breaks. From the remaining synteny blocks, the positions and alleles of all polymorphic sites were extracted, and their positions in the respective genomes recorded. A second Perl script was then applied to the recombinant genomes to determine their allelic composition at each polymorphic site.

To minimize errors or biases during mapping, sequencing reads were mapped simultaneously to both donor and recipient genomes using bwa [59]. Based on the best match, allele composition of each read was projected onto the genome alignment, and the allelic state at each polymorphic site was determined. This dual-mapping approach ensured an unbiased assignment of recombinant reads to donor or recipient origin.

Donor allele frequency was then calculated at each polymorphic site along the genome. To detect recombinant regions, we applied a stepwise segmentation analysis using the R package **«** changepoint **»** [61], with a penalty parameter of 3 to allow detection of small fragments.

All code is available on GitHub: https://github.com/J14567/HGT_2025.

### 4.10. Detection of integration sites

To confirm the genomic position of the marker under selection, we designed primers based on the inferred peak corresponding to the integration of the donor cassette (size ~2kb) into the recipient background. The first set of primers was placed approximately 1.5 kb upstream and downstream of the predicted locus. Additional primers within the integrated cassette were also designed. PCR amplification using the first set of primers successfully detected the integrated cassette at the predicted locus for 2 out of 3 samples. For the third sample, detection required pairing 1.5kb chromosomal primer with a cassette-specific primer (S4, S5A and S5B Figs). The PCR products ranging for 1 to 1.5kb were send for Sanger sequencing. Sequencing results were mapped to validate the presence of the marker and precisely identify the junction between the cassette and the chromosome. Finally, the experimentally obtained position was compared to the predicted location.

## Supporting information

S1 Table Strains. (XLSX)

S2 Table Plasmids. (XLSX)

S3 Table Primers. (XLSX)

S4 Table sgRNA sequences. (XLSX)

S5 Table Cas9-Lambda-red recombination-mediated Hfr construction. (XLSX)

S6 Table Validating the ability of constructed Hfr to transfer the galK marker. (XLSX)

S7 Table Testing of conjugation in galK- recipient backgrounds. (XLSX)

S8 Table Efficiency of the recombineering system with and without guide, with and without cas9. (XLSX)

S9 Table sgRNA efficiency tested in different Keio variants. (XLSX)

S10 Table MultiQC-report: general stats. (XLSX)

S11 Table MultiQC-report: sequence counts. (XLSX)

S12 Table Coverage information for all fastq. (XLSX)

S1 File Mathematical modeling. (PDF)

S1 Fig Design of the conjugative plasmid and efficiency of the Cas9-Lambda Red system. A) Schematics of the construction of the plasmid pNTM3_sgRNA. B) Boxplot represent the frequency of chromosomal insertion of the plasmid, calculated as the ratio of colony-forming units (CFU/mL) of transconjugant recombinants to CFU/mL of donor cells. Two *E. coli* strains were analyzed: the parental strain BW25113 and the *ybjm* knockout mutant from the Keio collection. In grey, cells were transformed with the control plasmid pNTM3_TetA_sacB_KmR (no gRNA). In orange, cells were transformed with pNTM3_sgRNA (sgRNA1), in which *sacB* is replaced by sgRNA1. Recombinants were selected and quantified on LB + tetracycline; the donors on LB + DAP + tetracycline.
(TIF)

S2 Fig Transposon sequencing of the Hfr donor libraries. A) Methodology for transposon sequencing. B) Relative Transposon Insertion Density along the genome plotted log2 fold increase of the coverage across the chromosome relative to the macrodomain Ter. Bins of 250kb have been used. Small red dots show the density of transposon insertion sites relative to the density of transposon insertion sites in the Ter macro-domain. Large blue dots include the sequencing coverage of the insertion sites, they represent the number of reads matching transposon insertions sites within a bin normalized by the number of reads matching transposon insertions sites within the Ter macro-domain. C) Correlation of the two previous metrics, the relative density of insertion sites (red dots in B) is plotted on the x axis and the relative coverage of insertion sites (blue dots in B) on the y axis. Dotted pink line shows the correlation between both variables. Both metrics evolve similarly, showing that the biases of insertion and coverage are linked. D) Barcoded presentation of the position of the 1140 independent insertion sites of the transposons along the genome.
(TIF)

S3 Fig Anti-bacterial activity assay. Serial dilutions illustrating the of anti-bacterial activity of some strains against K12::*ZeoR*. Each vertical line corresponds to the plating on Zeocin of 10µl drops of successive 10 fold dilutions of bacterial culture in which K12::ZeoR has been mixed with an alternative strain whose name is written on top of the column. Each strain was tested in two independent cultures. As all tested strains are Zeocin sensitive, the plating reveals therefore the density of K12::ZeoR in the mix. K12 is used as a control and lead to a high density of K12::ZeoR in the culture, while IAIA14 known for its toxicity to K12, eradicate almost fully K12::ZeoR as illustrated by the absence or very low density of K12::ZeoR. 536 decrease the density of K12:: ZeoR by three orders of magnitude while HS has no impact.
(TIF)

S4 Fig Comparison of the HMM method versus donor allelic frequencies method for recombinant analysis. Recombinant fragments obtained by the HMM method versus the method based on donor allele frequency. REL606 recombinants are on the left (dark blue line indicates *galK*). HS recombinants are on the middle plot (light blue line indicates *galK*). 536 recombinants are on the right plot (pink line indicates *galK*). Black boxes indicate recombinants which contain at least one recombined fragment detected by only one of the two methods. The vast majority of recombined fragments are detected by both methods with very similar limits. Yet, a few very short fragments, that were validated manually, are found exclusively by the allele frequency approach that can more easily detect very short recombined fragments. Statistics in the main text refer therefore to that method. Black arrows indicate the fragments detected by only one of the two methods.
(TIF)

S5 Fig Impact of coverage on detection of recombination. The plot shows the number detected recombined fragments (y-axis) as a function of the log10 of the sequencing coverage used in the recombinant fastq file (x-axis). The data are derived from the 8 536 recombinants that were sequenced with high coverage. For each of these recombinants, a yellow dot shows the number of transfers detected at the initial coverage. These points are then connected to dots that shift their colour to blue and report the number of recombined fragments found when coverage used to detect the recombinants was artificially decreased with tool seqtk to 50%, 40%, 25%, 17.5% or 10% of the initial value. Each line represent therefore how coverage affects the detection of recombined fragments. Below a coverage of 5, some fragments are not detected. The histograms show the coverage of the sequenced recombinant for REL606 (dark blue) HS (light blue) and 536 (pink). The coverage of the recombinant is in a zone where detection of recombinant is robust.
(TIFF)

S6 Fig Genetic design of the primers to identify by PCR the Tse2 cassette integrated sites in the mutants. Schematics representing primer design for the identification of the Tse2 cassette insertion site from the predicted position of the cassette.
(TIF)

S7 Fig Agarose gel of PCR amplified Tse2 cassette integrated into recombinants. A) Agarose gel showing PCR amplification products to verify the insertion of the 3 kb Tse2 cassette into the genome. Three REL606 clones (noted as L, M and N) containing the cassette Tse2 were tested, each in replicate, with the REL606 ancestral strain as a control. PCR primers are listed in the S3 Table. The following sets were used for the clones: L_pool_F/ L_pool_R for clone L, M_pool_F/ M_pool_R for clone M, the following three sets: N_pool_F/ N_pool_R and N_F_before/N_R_before and N_F_after/N_R_after were used for clone N. The sets of primers were used for REL606 as a negative control. A band at 5 kb indicates successful insertion of the cassette – 3 kb of the cassette + the 2kb of the upstream and downstream of the chromosome - while a band at 2 kb indicates the absence of insertion -only the chromosome was amplified. No PCR product was observed for clone N (using the pair of primers N_pool_F/ N_pool_R), indicating that the targeted region was not within the expected interval. To resolve this, new primers targeting a larger region flanking the insertion site (N_F_before/N_R_before and N_F_after/N_R_after) with a 1.5 kb resolution, respectively upstream and downstream the peak location.B) PCR verification of cassette orientation and presence in clone L and N using a different set of primers (L_pool_F/Tse2_F (1), L_pool_R/Tse2_R (2), N_F_before/Tse2_F(3) and N_R_before/Tse2_R (4)). The clones L and N are on the left side of the gel and the control on the right side. The same sets of primers were tested for the controls. PCR bands for the successfully amplified clones with the reverse primers’ sets were extracted and sent for DNA sequencing. Results confirmed correct cassette insertion in both clones and its absence in the controls.
(TIF)

S8 Fig Circular Alignment of the donor K-12 against the recipients REL606, HS and 536. The alignment was performed on Brig using K12 as a reference. The black arrow indicates the position of *galK.*
(TIF)

S9 Fig Density of opportunities for recombination initiation along the chromosome. Each track of 20 bp (lower bound) or 27 bp (upper bound) showing strict homology between donor and recipient can be used to initiate homologous recombination. Given the low divergence between strains, when we average the number of these opportunities for recombination per kb across bins of 100kb for each of the tree recipients (REL606, HS and 536), we see that focusing on the 700kb before and after *galK* locus there are several hundred of opportunities for recombination per kb. The numbers decrease from REL606–536, but remain high.
(TIF)
