# Supplementary material for: High-throughput conjugation reveals strain specific recombination patterns enabling precise trait mapping in Escherichia coli
Source: PLoS Genet. 2025 Oct 30;21(10):e1011636. doi: 10.1371/journal.pgen.1011636 (PMC12594344; doi:10.1371/journal.pgen.1011636)
Supplement: S1 File — (PDF) [file pgen.1011636.s013.pdf]

Maximal recombined interval straddling 0

Amaury Lambert

March 12, 2024

1 Introduction and notation

We consider a genomic sequence modeled by a continuous segment or line and are specifically interested in a core allele located at position 0 on the line. We assume that $I_1, I_2, \ldots, I_n$ are n random, independent intervals straddling 0 and modeling the span of n independent crossover events. We are specifically interested in the conserved region containing the core allele, namely the interval

$$(\Gamma, \Delta) := I_1 \cap I_2 \cap \cdots \cap I_n.$$

The length of this maximal conserved interval is denoted

$$\Lambda := \Delta - \Gamma.$$

With the notation $I_j = (G_j, D_j)$, we thus have

$$\Gamma = \max(G_1, ..., G_n) \quad \text{and} \quad \Delta = \min(D_1, ..., D_n). \tag{1}$$

From now on, we will use the following notation:

- For any event A, $\mathbb{1}_A$ denotes the Bernoulli random variable which takes the value 1 if A is realized and 0 otherwise;
- For any real number x, the notation "dx" both denotes the length of an infinitesimal interval around x and the interval itself, in such a way that for any random variable X with density f, $P(X \in dx)$ stands for $P(X \in [x, x+dx))$ and is equal to $f(x)\,dx$.

The following equation is a straightforward consequence of (1) that we will use repeatedly in this note. For all $x < 0 < y$,

$$\begin{aligned} P(\Gamma \in dx, \Delta \in dy) &= \sum_i \sum_{j \neq i} P(G_i \in dx, D_i > y) P(D_j \in dy, G_j < x) \prod_{k \neq i,j} P(G_k < x, D_k > y) \\ &+ \sum_i P(G_i \in dx, D_i \in dy) \prod_{k \neq i} P(G_k < x, D_k > y). \end{aligned} \tag{2}$$

We define three Assumptions labelled from 1 to 3 as follows:

1. For each i, conditional on $D_i - G_i = \ell_i$ and setting $U_i := -G_i/\ell_i$, so that $(G_i, D_i) = (-U_i\ell_i, (1-U_i)\ell_i)$, U_i **is uniformly distributed in** $(0, 1)$;

2. All random pairs (G_i, D_i) have the same law as some random pair (G, D) and so are iid, that is, (not only) independent (but also) **identically distributed**. In addition, the random variable $L := D - G$ has a continuous density denoted h.

3. The density h can be specified in terms of a parent density p which is the density of the length Y of a typical recombining interval, while h **is the density of the length** L **of a typical recombining interval conditioned to straddle** 0. If we assume that a typical recombining interval has finite expected length $m := E(Y) = \int_0^\infty yp(y)\,dy$, then it is well-known that Assumption 1 holds and that
$$h(y) = \frac{yp(y)}{m} \qquad y > 0,$$
called the **size-biased density of** Y, which translates mathematically the intuition that the intervals straddling 0 (or straddling any position on the line that the observer has fixed in advance) are statistically larger than other intervals (the so-called 'waiting time paradox').

Combining the results of forthcoming Propositions 3.3 and 4.1, we get the following

Theorem 1.1. *We assume that Assumptions 1 and 2 are in force. The maximal conserved interval* (Γ, Δ) *has the law of* $(-U\Lambda, (1-U)\Lambda)$*, where* U *is independent of* Λ *and uniform in (0,1), and the law of* Λ *is given by*

$$P(\Lambda > z) = nzf(z)F(z)^{n-1} + F(z)^n \qquad z > 0,$$

where f *is the density of* D *and* $F(z) = P(D > z)$*. In addition, the functions* f *and* F *can be expressed in terms of the density* h *of* L *as follows*

$$f(z) = \int_z^\infty \frac{h(u)}{u}\,du$$

and
$$F(z) = \int_z^\infty f(x)\,dx = \int_z^\infty \frac{(u-z)}{u}h(u)\,du = \int_z^\infty h(u)\,du - zf(z).$$
In the case when $L = \ell > 0$ *is not random,*

$$E(\Lambda) = \frac{2\ell}{n+1} \qquad \text{and} \qquad Var(\Lambda) = \frac{2(n-1)\ell^2}{(n+1)^2(n+2)}.$$

Next assume $E(1/L)$ *is finite and set* $\mu := 1/E(1/L)$*. Then the pairs* $(n\Gamma_n, n\Delta_n)$ *(we have added a subscript* n *to recall that* n *varies) converge in law as* $n \to \infty$ *to* $(-\mu V, \mu W)$*, where* V *and* W *are two independent exponential random variables with parameter 1. Last, if Assumption 3 is in force, then* $E(1/L)$ *is finite and* $\mu = m$*.*

2 Intervals with fixed lengths

Here we condition on the lengths $\ell_1, \ell_2, \dots, \ell_n$ of the recombining intervals $I_1, I_2, \dots, I_n$, so that $D_i - G_i = \ell_i$ for all i. We assume that Assumption 1 is in force, that is, the intervals are positioned in such a way that from the point of view of an interval, 0 can be seen as independently, uniformly located within the interval. Otherwise said, for each i, $(G_i, D_i) = (-U\ell_i, (1-U)\ell_i)$, where U is uniformly distributed in $(0,1)$.

Under this assumption the G_i's (and D_i's also) are pairwise distinct, so there is a unique pair (i,j) such that $\Gamma = G_i$ and $\Delta = D_j$. In the right-hand side of the following equation, the first term corresponds to the case when $i \neq j$ and the second term to the case when $i = j$. Using Equation (2) and the independence of the interval positions, we get for all $x < 0 < y$,

$$\begin{aligned} P(\Gamma \in dx, \Delta \in dy) &= \sum_i \sum_{j \neq i} \frac{dx}{\ell_i} \mathbb{1}_{\ell_i > y-x} \frac{dy}{\ell_j} \mathbb{1}_{\ell_j > y-x} \prod_{k \neq i,j} \mathbb{1}_{\ell_k > y-x} \frac{\ell_k - (y-x)}{\ell_k} \\ &+ \sum_i \mathbb{1}_{\ell_i = y-x} \frac{dx}{y-x} \prod_{k \neq i} \mathbb{1}_{\ell_k > y-x} \frac{\ell_k - (y-x)}{\ell_k}. \end{aligned} \tag{3}$$

3 Intervals with iid lengths

From now on, Assumptions 1 and 2 are in force. Recall that the maximal conserved interval, defined as the intersection of $I_1, I_2, \dots, I_n$, is denoted (Γ, Δ) and its length is denoted $\Lambda = \Delta - \Gamma$. Then a first straightforward consequence of Equation (2) and Assumption 2 is

$$\begin{aligned} P(\Gamma \in dx, \Delta \in dy) &= n(n-1) P(G \in dx, D > y) P(D \in dy, G < x) P(G < x, D > y)^{n-2} \\ &+ n P(G \in dx, D \in dy) P(G < x, D > y)^{n-1} \qquad x < 0 < y. \end{aligned}$$

Now because of Assumptions 1 and 2, we get

$$P(G \in dx, D \in dy) = \frac{h(y-x)}{y-x}\, dx\, dy \qquad x < 0 < y$$

or equivalently (recall $L = D - G$)

$$P(L \in dz, D \in dy) = \frac{h(z)}{z}\, dy\, dz \qquad 0 < y < z.$$

Lemma 3.1. *For any $z > 0$, set*

$$f(z) := E\left(\frac{\mathbb{1}_{L>z}}{L}\right) = \int_z^\infty \frac{h(u)}{u}\, du$$

and $F(z) := \int_z^\infty f(x)\, dx$. Then f is the density of D and

$$F(z) = P(D > z) = E\left(\frac{(L-z)}{L} \mathbb{1}_{L>z}\right) = \int_z^\infty \frac{(u-z)}{u} h(u)\, du = \int_z^\infty h(u)\, du - z f(z).$$

In addition,

$$\lim_{z\to\infty} f(z) = 0 \quad \text{and} \quad \lim_{z\to 0} f(z) = E\left(\frac{1}{L}\right),$$

which may be finite or infinite depending on the the law of L. Also,

$$\lim_{z\to 0} F(z) = 1 \quad \text{and} \quad \lim_{z\to\infty} F(z) = 0.$$

Remark 3.2. *Under Assumption 3, that is, when h is the size-biased density of the random variable Y with density p, i.e., $h(y) = yp(y)/m$, where $m := \int_0^\infty yp(y)\,dy$ is assumed to be finite, we have for any $z > 0$,*

$$f(z) = m^{-1}\int_z^\infty p(u)\,du = m^{-1}P(Y > z)$$

and

$$F(z) = m^{-1}\int_z^\infty (u-z)p(u)\,du = m^{-1}E\left((Y-z)\mathbb{1}_{Y>z}\right).$$

In addition,

$$E\left(\frac{1}{L}\right) = \int_0^\infty \frac{h(u)}{u}\,du = m^{-1}\int_0^\infty p(u)\,du = \frac{1}{m}.$$

Proof. As noted before stating Lemma 3.1, $P(L \in dz, D \in dy) = \frac{h(z)}{z}\,dy\,dz$, so that

$$P(D \in dy) = \left(\int_y^\infty \frac{h(z)}{z}\,dz\right)dy = E\left(\frac{\mathbb{1}_{L>y}}{L}\right)dy \qquad y > 0,$$

and

$$P(D > z) = \int_z^\infty E\left(\frac{\mathbb{1}_{L>y}}{L}\right)dy \qquad y > 0,$$

which, by Fubini–Tonelli Theorem equals

$$P(D > z) = E\left(\frac{1}{L}\int_z^\infty \mathbb{1}_{y<L})\,dy\right) = E\left(\frac{(L-z)\mathbb{1}_{L>z}}{L}\right) \qquad z > 0.$$

The other equalities are straightfoward. Now let us prove the four limits. First, $f(z) \leq P(D > z)/z$ and so goes to 0 as $z \to \infty$. Second, for any nonincreasing sequence (z_k) converging to 0 as $k \to \infty$, the non-negative random variables $(\mathbb{1}_{L>z_k}/L)$ form a nondecreasing sequence converging to $1/L$ so by the Monotone Convergence Theorem,

$$\lim_{k\to\infty} f(z_k) = \lim_{k\to\infty} E(\mathbb{1}_{L>z_k}/L) = E(\lim_{k\to\infty} \mathbb{1}_{L>z_k}/L) = E(1/L).$$

Finally, the last two limits hold because $F(z) = P(D > z)$. □

The following proposition characterizes the law of (Γ, Δ) using the notation introduced in the previous lemma.

Proposition 3.3. *We have*

$$P(\Lambda > z) = nzf(z)F(z)^{n-1} + F(z)^n \qquad z > 0,$$

and conditional on $\Lambda = z$, Δ is uniformly distributed in $(0, z)$.

In particular,

$$E(\Lambda) = 2\int_0^\infty F(x)^n dx \quad \text{and} \quad E(\Lambda^2) = \int_0^\infty 6xF(x)^n dx. \tag{4}$$

In the case when L is not random and takes the fixed value ℓ,

$$P(\Lambda > x) = n(x/\ell)(1 - x/\ell)^{n-1} + (1 - x/\ell)^n \qquad 0 < x < \ell,$$

and $P(\Lambda > x) = 0$ if $x \geq \ell$. Furthermore,

$$E(\Lambda) = \frac{2\ell}{n+1} \quad \text{and} \quad Var(\Lambda) = \frac{2(n-1)\ell^2}{(n+1)^2(n+2)}.$$

Proof. Using Equation (3) and integrating it over the values of the interval lengths ℓ_i's, we get for any $x < 0 < y$,

$$\begin{aligned} P(\Gamma \in dx, \Delta \in dy) &= n(n-1)f(y-x)^2 F(y-x)^{n-2}\, dx\, dy \\ &+ n(h(y-x)/(y-x))F(y-x)^{n-1}\, dx\, dy. \end{aligned} \tag{5}$$

Equivalently, for any $0 < y < z$,

$$P(\Lambda \in dz, \Delta \in dy) = n(n-1)f(z)^2 F(z)^{n-2}\, dy\, dz + n(h(z)/(z))F(z)^{n-1}\, dy\, dz. \tag{6}$$

Integrating over $y \in (0, z)$ in the last equation yields

$$P(\Lambda \in dz) = n(n-1)zf(z)^2 F(z)^{n-2}\, dz + nh(z)F(z)^{n-1}\, dz,$$

so that using (6), we see that conditional on $\Lambda = z$, Δ is uniformly distributed in $(0, z)$.

Now notice that f and F are differentiable in $(0, \infty)$ and that for any $z > 0$,

$$f'(z) = -h(z)/z \quad \text{and} \quad F'(z) = -f(z).$$

As a consequence, we can integrate by parts in the following equation (using limits stated in Lemma 3.1)

$$\begin{aligned} P(\Lambda > x) &= \int_x^\infty (n(n-1)zf(z)^2 F(z)^{n-2} + nh(z)F(z)^{n-1})dz \\ &= [-nzf(z)F(z)^{n-1}]_x^\infty + \int_x^\infty (n(zf'(z) + f(z))F(z)^{n-1} + nh(z)F(z)^{n-1})dz \\ &= nxf(x)F(x)^{n-1} + \int_x^\infty nf(z)F(z)^{n-1}dz \\ &= nxf(x)F(x)^{n-1} + [-F(z)^n]_x^\infty \\ &= nxf(x)F(x)^{n-1} + F(x)^n. \end{aligned}$$

Recall the formula $E(\Lambda)=\int_0^\infty P(\Lambda>x)\,dx$, which yields, by an integration by parts

$$\begin{aligned} E(\Lambda) &= [-xF(x)^n]_0^\infty + 2\int_0^\infty F(x)^n dx \\ &= 2\int_0^\infty F(x)^n dx. \end{aligned}$$

Note that we could have obtained the preceding expression by noting that $P(\Delta>x)=P(D>x)^n$ and that $E(\Lambda)=E(\Delta)-E(\Gamma)=2E(\Delta)$, because Δ and $-\Gamma$ have the same law.

Similarly,

$$\begin{aligned} E(\Lambda^2) &= \int_0^\infty 2xP(\Lambda>x)dx \\ &= [-2x^2F(x)^n]_0^\infty + \int_0^\infty 4xF(x)^n dx + \int_0^\infty 2xF(x)^n dx \\ &= \int_0^\infty 6xF(x)^n dx. \end{aligned}$$

In the case when L is not random and takes the fixed value ℓ, $F(x)=1-x/\ell$ if $x<\ell$ and $F(x)=0$ otherwise. Also, $f(x)=1/\ell$ if $x<\ell$ and $f(x)=0$ otherwise. Then for any $0<x<\ell$,

$$P(\Lambda>x)=n(x/\ell)(1-x/\ell)^{n-1}+(1-x/\ell)^n,$$

and $P(\Lambda>x)=0$ if $x\geq\ell$. Then we get

$$E(\Lambda)=2\int_0^\infty F(x)^n dx = 2\int_0^\ell (1-x/\ell)^n dx = 2\ell/(n+1)$$

and

$$\begin{aligned} E(\Lambda^2) &= \int_0^\infty 6xF(x)^n dx = \int_0^\ell 6x(1-x/\ell)^n dx \\ &= \ell\int_0^1 6\ell u(1-u)^n du = 6\ell^2\int_0^1 (1-v)v^n dv = 6\ell^2(1/(n+1)-1/(n+2)) \\ &= 6\ell^2/(n+1)(n+2). \end{aligned}$$

As a consequence,

$$\begin{aligned} \mathrm{Var}(\Lambda) &= E(\Lambda^2)-E(\Lambda)^2 = 6\ell^2/(n+1)(n+2) - 4\ell^2/(n+1)^2 \\ &= [2\ell^2/(n+1)][3/(n+2)-2/(n+1)] = 2\ell^2(n-1)/(n+2)(n+1)^2, \end{aligned}$$

which ends the proof. □

4 Large n asymptotic

Here, we seek to study the behavior of the maximal conserved interval as the number n of recombining intervals straddling 0 gets large. We denote by (Γ_n, Δ_n) this interval and by Λ_n its length, in order to recall the dependence on n. We know that as n gets large Λ_n vanishes. Our goal is to characterize the speed at which Λ_n goes to 0 and its limit once correctly renormalized, under Assumptions 1 and 2 stated in Introduction (iid interval lengths and uniform position of 0 within each interval, independently).

Proposition 4.1. *Assume that the typical length L of a recombining interval satisfies*

$$E\left(\frac{1}{L}\right) < \infty,$$

and set

$$\mu := \frac{1}{E\left(\frac{1}{L}\right)}$$

its "harmonic expectation".

Then $(n\Gamma_n, n\Delta_n)$ converges in law as $n \to \infty$ to the random pair $(-\mu V, \mu W)$, where V and W are two independent exponential random variables with parameter 1.

In particular, $n\Lambda_n$ converges in law to the random variable μZ where Z has density g given by $g(z) = ze^{-z}$ for $z > 0$.

This convergence extends to the moments of Λ_n as follows

$$\lim_{n\to\infty} E(n\Lambda_n) = \mu E(Z) = 2\mu,$$

and

$$\lim_{n\to\infty} Var(n\Lambda_n) = \mu^2\, Var(Z) = 2\mu^2.$$

Remark 4.2. *The asymptotic independence between Γ_n and Δ_n is due to the fact that it becomes increasingly unlikely as n gets large that $(\Gamma_n, \Delta_n) = (G_i, D_i)$ for the same $i \in \{1, \ldots, n\}$. Then, with high probability, $\Gamma_n = G_i$ and $\Delta_n = D_j$ for some $i \neq j$, so they can be considered as the extremes of two independent sets of iid random variables. The convergence to the exponential distribution is a well-known result of extreme value theory.*

Remark 4.3. *As noted in Remark 3.2, in the case when h is the size-biased density of some random variable Y with density p (Assumption 3), i.e., $h(y) = yp(y)/m$, where $m = E(Y)$, then*

$$\mu = m,$$

that is, when L follows the size-biased distribution of Y, the harmonic expectation of L equals the expectation of Y.

We first state a useful lemma.

Lemma 4.4. *Recall the random pair (V, W) and the random variable Z in Proposition 4.1. The random pair (V, W) has the same law as $(UZ, (1-U)Z)$, where U is uniform in $(0, 1)$ and independent of Z.*

Proof. We show that the bivariate Laplace transforms of the two pairs are equal. For any distinct $x, y > 0$,

$$\begin{aligned}
E(\exp(-xUZ - y(1-U)Z)) &= \int_0^1 du \int_0^\infty e^{-xuz-y(1-u)z} ze^{-z} dz \\
&= \int_0^\infty \left(\int_0^1 e^{-xuz+yuz} du \right) ze^{-yz-z} dz \\
&= \int_0^\infty \left[\frac{e^{(-xz+yz)u}}{-xz+yz} \right]_0^1 ze^{-yz-z} dz \\
&= \int_0^\infty \frac{e^{-xz+yz} - 1}{-xz+yz} ze^{-yz-z} dz \\
&= \frac{1}{-x+y} \int_0^\infty \left(e^{-xz+yz} - 1 \right) e^{-yz-z} dz \\
&= \frac{1}{-x+y} \int_0^\infty \left(e^{-xz-z} - e^{-yz-z} \right) dz \\
&= \frac{1}{-x+y} \left(\frac{1}{x+1} - \frac{1}{y+1} \right) \\
&= \frac{1}{(x+1)(y+1)}.
\end{aligned}$$

Now

$$\begin{aligned}
E(\exp(-xV - yW)) &= E(\exp(-xV))E(\exp(-yW)) \\
&= \int_0^\infty e^{-xv} e^{-v} dv \int_0^\infty e^{-yw} e^{-w} dw = \frac{1}{(x+1)(y+1)},
\end{aligned}$$

which ends the proof. □

Proof of Proposition 4.1. Recall from Proposition 3.3 that conditional on $\Lambda_n = z$, Δ_n is uniformly distributed in $(0, z)$. In other words, (Γ_n, Δ_n) has the same law as $(-U\Lambda_n, (1-U)\Lambda_n)$, where U is uniform in $(0, 1)$ and independent of Λ_n. If (we manage to prove that) $n\Lambda_n$ converges to μZ, then obviously (Γ_n, Δ_n) converges to $\mu(-UZ, (1-U)Z)$, where U is uniform and independent of Z, and thanks to Lemma 4.4, we have proved the convergence of (Γ_n, Δ_n) to $\mu(-V, W)$.

Now recall from Proposition 3.3 that

$$P(\Lambda_n > z) = nzf(z)F(z)^{n-1} + F(z)^n \qquad z \geq 0,$$

so that

$$P(n\Lambda_n > z) = zf(z/n)F(z/n)^{n-1} + F(z/n)^n \qquad z \geq 0,$$

Now because $E(1/L)$ is finite, we know from Lemma 3.1 that f has a limit at 0+ and that F is differentiable at 0+ with $F'(0+) = -f(0+) = c$ with $c = E(1/L)$. Then we have $F(z/n) = 1 - cz/n + o(1/n)$ and it is easily seen that

$$\lim_{n\to\infty} P(n\Lambda_n > z) = zce^{-cz} + e^{-cz} \qquad z > 0,$$

which can also be expressed as

$$\lim_{n\to\infty} P(cn\Lambda_n > z) = ze^{-z} + e^{-z} \qquad z > 0.$$

Now if Z is a random variable with density $g(z) = ze^{-z}$, then $P(Z > z) = ze^{-z} + e^{-z}$, so that

$$\lim_{n\to\infty} P(cn\Lambda_n > z) = P(Z > z) \qquad z > 0,$$

and finally

$$\lim_{n\to\infty} P(n\Lambda_n > z) = P(Z/c > z) \qquad z > 0,$$

which proves that $n\Lambda_n$ converges in law to Z/c, which ends the proof of convergence since $\mu = 1/c$.

Convergence of the moments can be obtained by using Equation (4), the change of variable $x = z/n$ and standard truncation arguments. $\square$
