# Supplementary figures and images for: High-throughput conjugation reveals strain specific recombination patterns enabling precise trait mapping in Escherichia coli
Source: PLoS Genet. 2025 Oct 30;21(10):e1011636. doi: 10.1371/journal.pgen.1011636 (PMC12594344; doi:10.1371/journal.pgen.1011636)

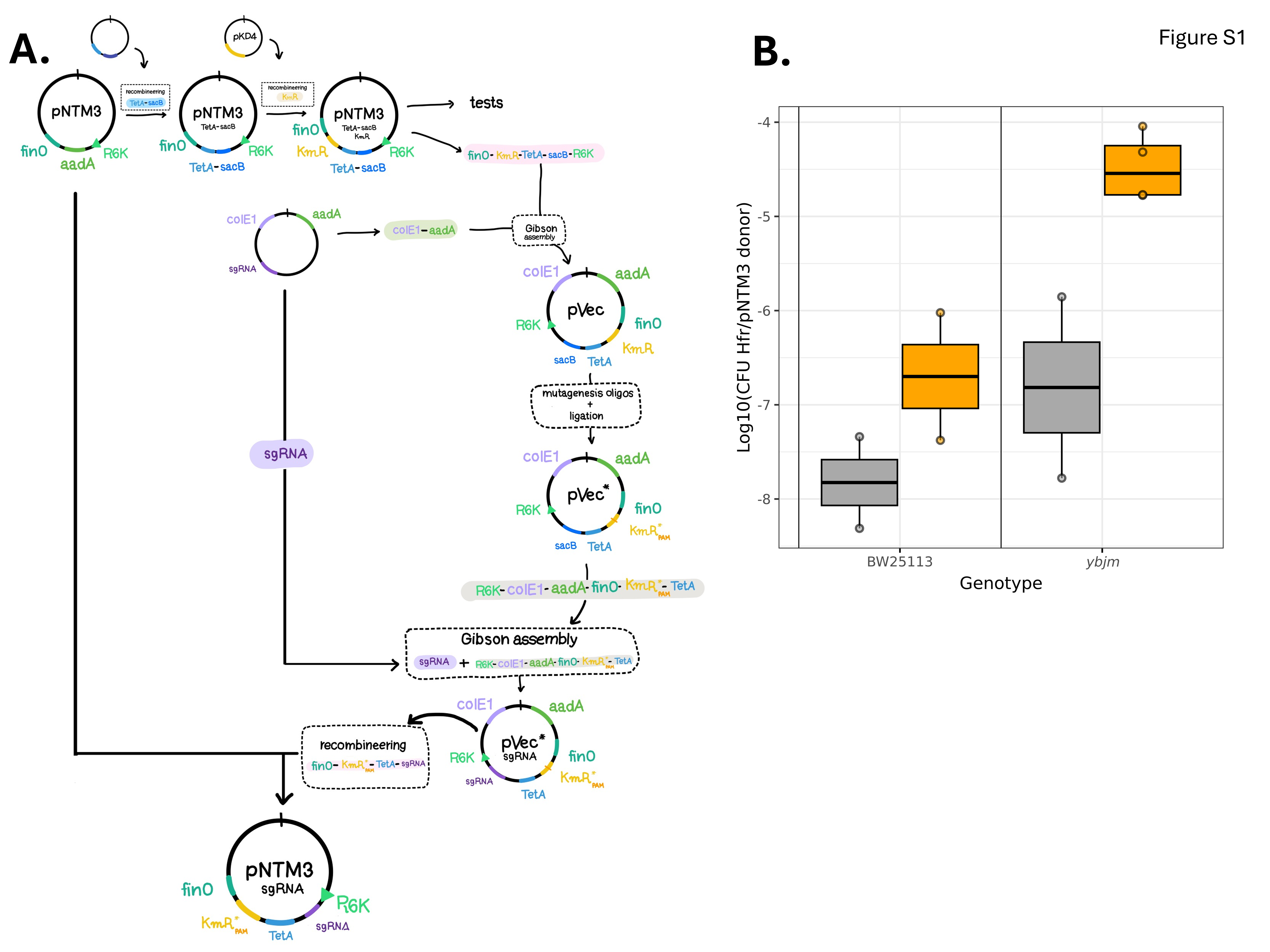

Supplement: S1 Fig — A) Schematics of the construction of the plasmid pNTM3_sgRNA. B) Boxplot represent the frequency of chromosomal insertion of the plasmid, calculated as the ratio of colony-forming units (CFU/mL) of transconjugant recombinants to CFU/mL of donor cells. Two E. coli strains were analyzed: the parental strain BW25113 and the ybjm knockout mutant from the Keio collection. In grey, cells were transformed with the control plasmid pNTM3_TetA_sacB_KmR (no gRNA). In orange, cells were transformed with pNTM3_sgRNA (sgRNA1), in which sacB is replaced by sgRNA1. Recombinants were selected and quantified on LB + tetracycline; the donors on LB + DAP + tetracycline. (TIF) [file pgen.1011636.s014.TIF]

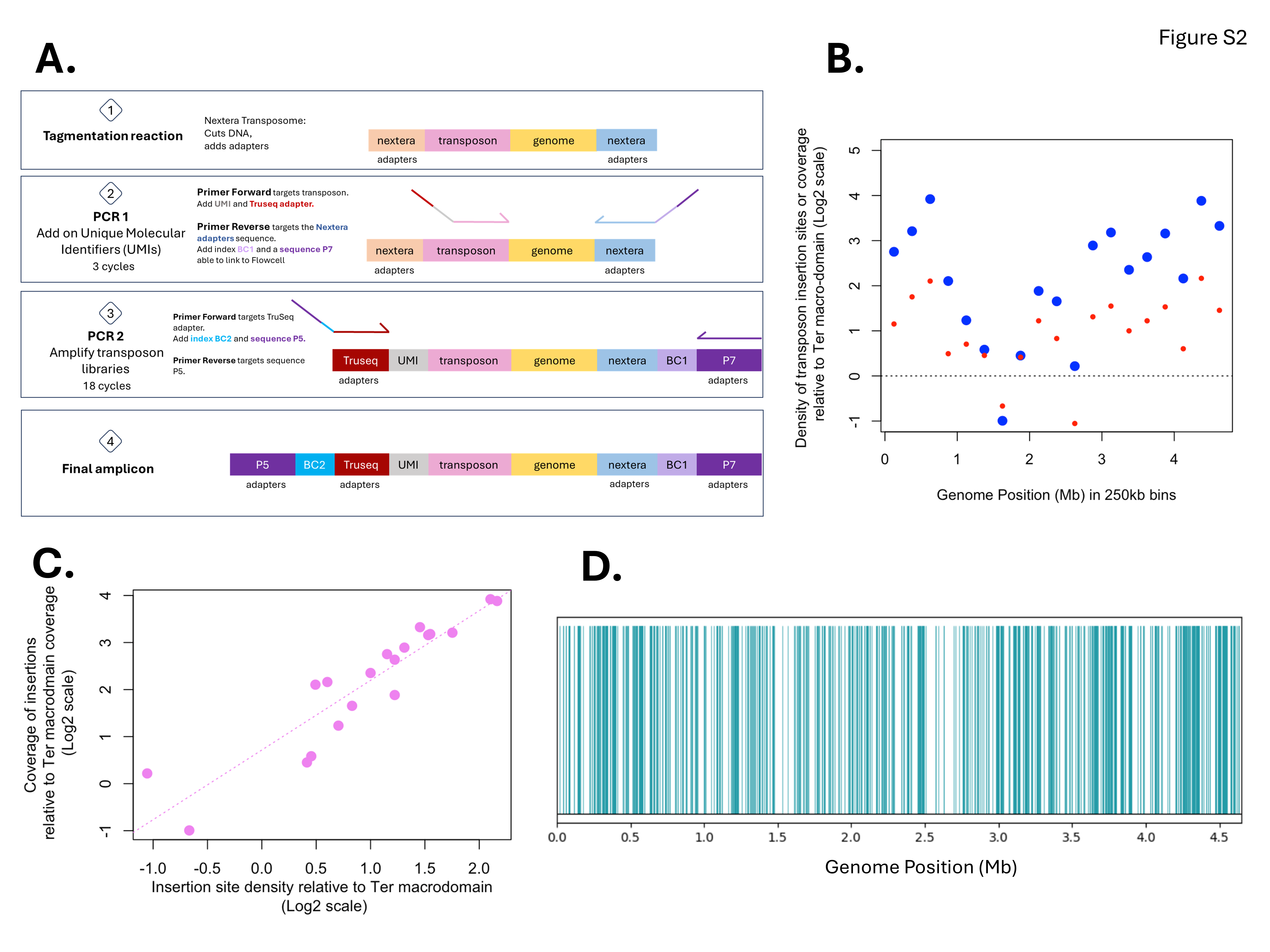

Supplement: S2 Fig — A) Methodology for transposon sequencing. B) Relative Transposon Insertion Density along the genome plotted log2 fold increase of the coverage across the chromosome relative to the macrodomain Ter. Bins of 250kb have been used. Small red dots show the density of transposon insertion sites relative to the density of transposon insertion sites in the Ter macro-domain. Large blue dots include the sequencing coverage of the insertion sites, they represent the number of reads matching transposon insertions sites within a bin normalized by the number of reads matching transposon insertions sites within the Ter macro-domain. C) Correlation of the two previous metrics, the relative density of insertion sites (red dots in B) is plotted on the x axis and the relative coverage of insertion sites (blue dots in B) on the y axis. Dotted pink line shows the correlation between both variables. Both metrics evolve similarly, showing that the biases of insertion and coverage are linked. D) Barcoded presentation of the position of the 1140 independent insertion sites of the transposons along the genome. (TIF) [file pgen.1011636.s015.TIF]

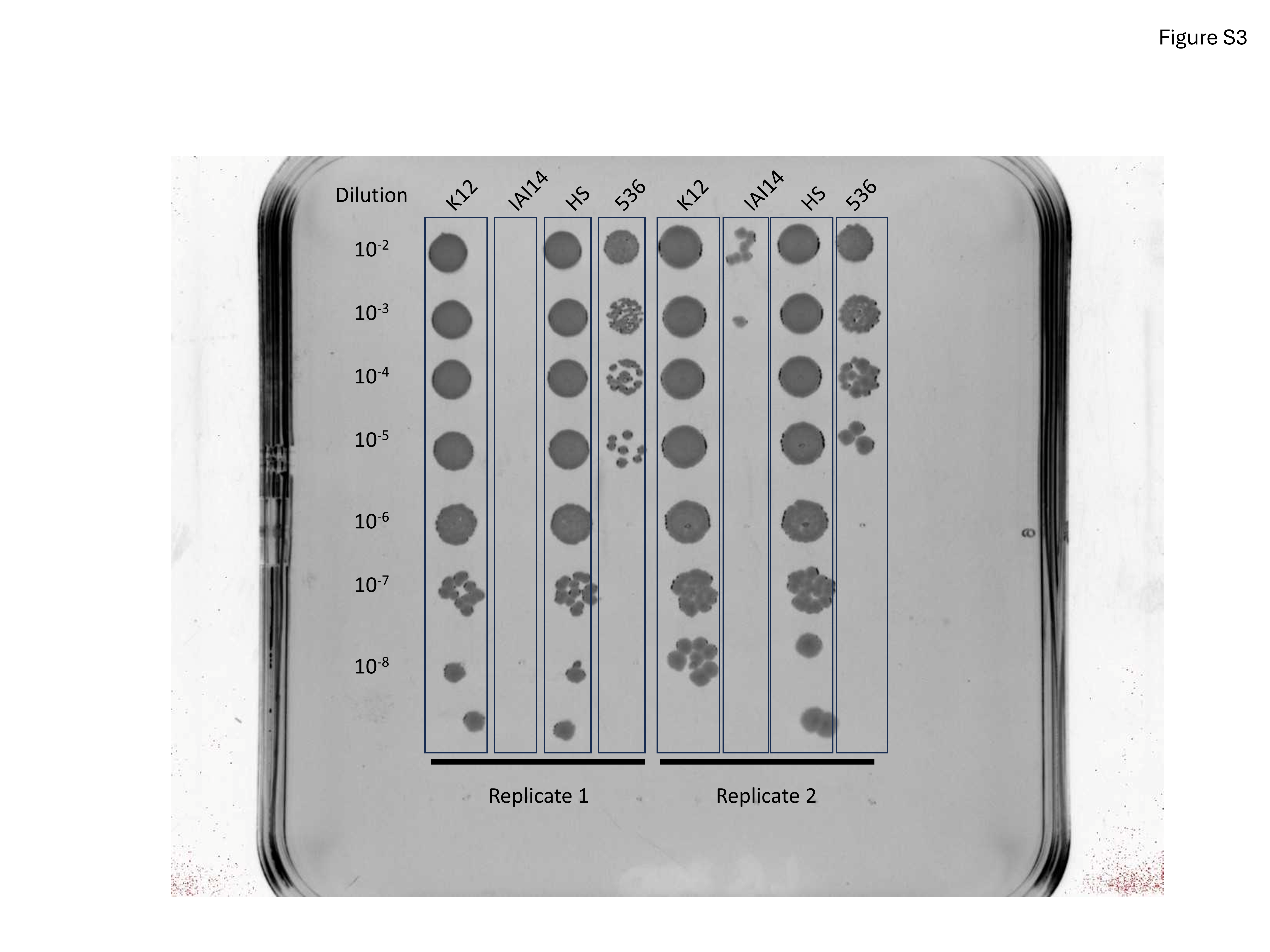

Supplement: S3 Fig — Serial dilutions illustrating the of anti-bacterial activity of some strains against K12::ZeoR. Each vertical line corresponds to the plating on Zeocin of 10µl drops of successive 10 fold dilutions of bacterial culture in which K12::ZeoR has been mixed with an alternative strain whose name is written on top of the column. Each strain was tested in two independent cultures. As all tested strains are Zeocin sensitive, the plating reveals therefore the density of K12::ZeoR in the mix. K12 is used as a control and lead to a high density of K12::ZeoR in the culture, while IAIA14 known for its toxicity to K12, eradicate almost fully K12::ZeoR as illustrated by the absence or very low density of K12::ZeoR. 536 decrease the density of K12:: ZeoR by three orders of magnitude while HS has no impact. (TIF) [file pgen.1011636.s016.TIF]

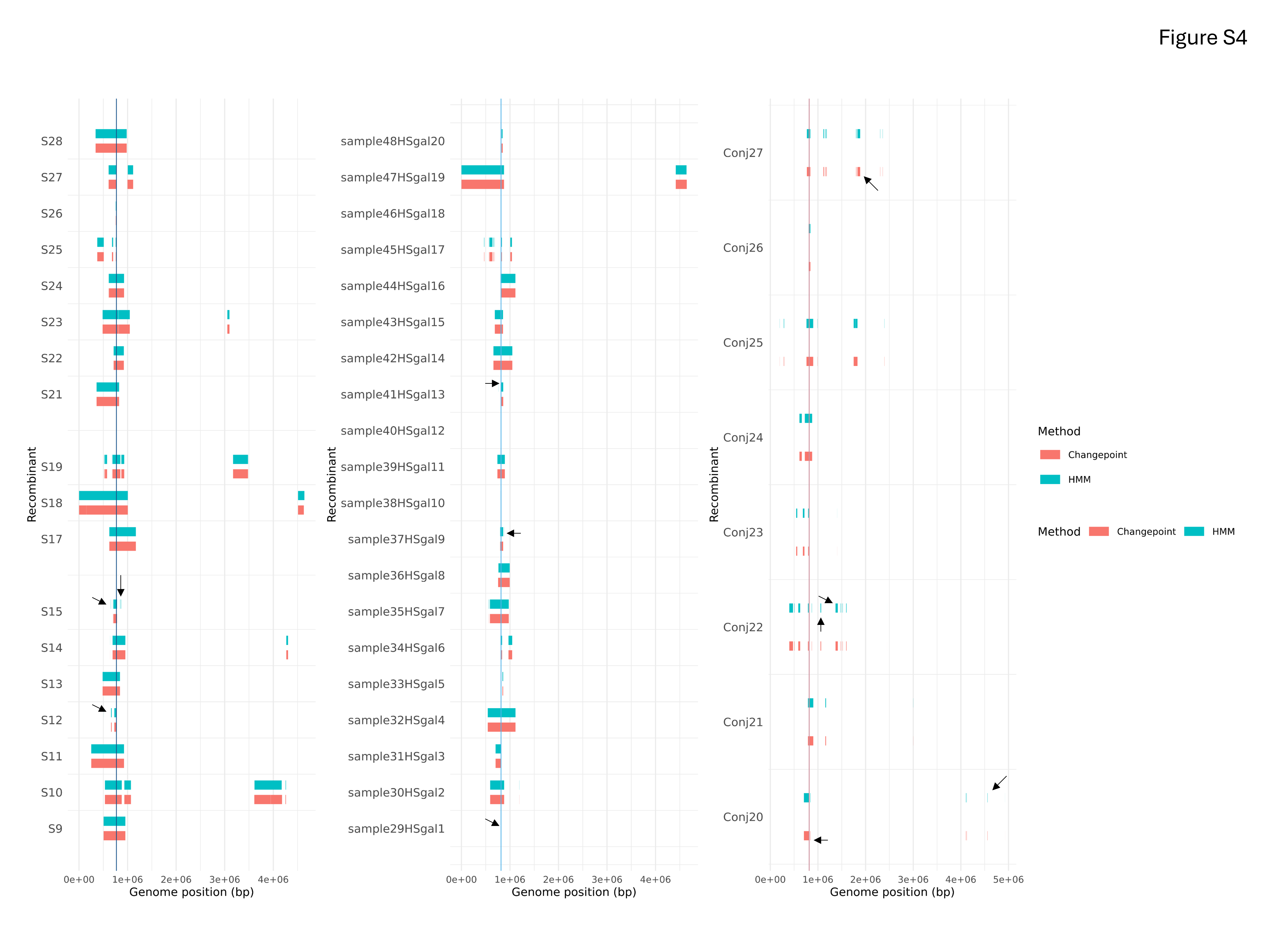

Supplement: S4 Fig — Recombinant fragments obtained by the HMM method versus the method based on donor allele frequency. REL606 recombinants are on the left (dark blue line indicates galK). HS recombinants are on the middle plot (light blue line indicates galK). 536 recombinants are on the right plot (pink line indicates galK). Black boxes indicate recombinants which contain at least one recombined fragment detected by only one of the two methods. The vast majority of recombined fragments are detected by both methods with very similar limits. Yet, a few very short fragments, that were validated manually, are found exclusively by the allele frequency approach that can more easily detect very short recombined fragments. Statistics in the main text refer therefore to that method. Black arrows indicate the fragments detected by only one of the two methods. (TIF) [file pgen.1011636.s017.TIF]

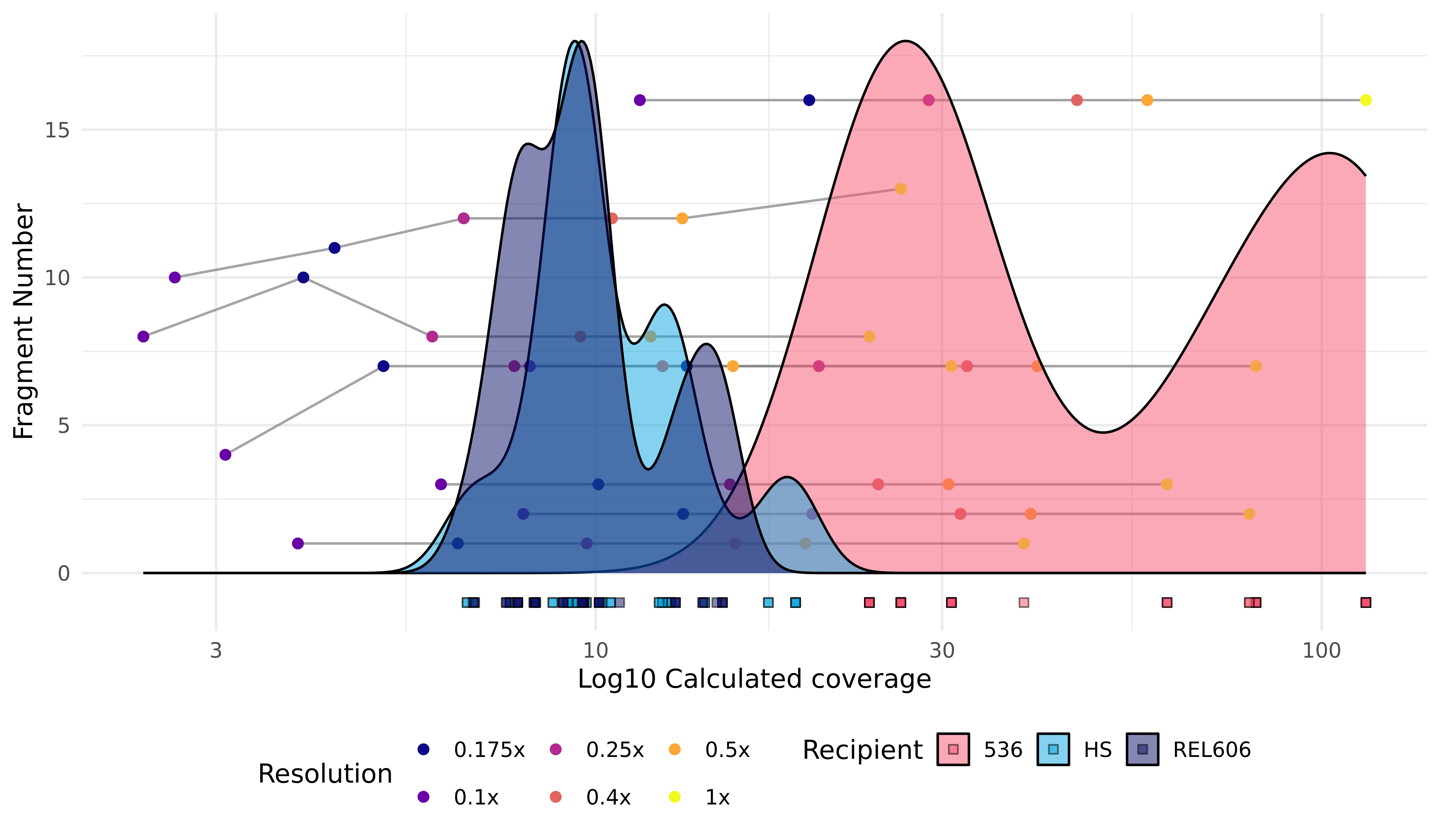

Supplement: S5 Fig — The plot shows the number detected recombined fragments (y-axis) as a function of the log10 of the sequencing coverage used in the recombinant fastq file (x-axis). The data are derived from the 8 536 recombinants that were sequenced with high coverage. For each of these recombinants, a yellow dot shows the number of transfers detected at the initial coverage. These points are then connected to dots that shift their colour to blue and report the number of recombined fragments found when coverage used to detect the recombinants was artificially decreased with tool seqtk to 50%, 40%, 25%, 17.5% or 10% of the initial value. Each line represent therefore how coverage affects the detection of recombined fragments. Below a coverage of 5, some fragments are not detected. The histograms show the coverage of the sequenced recombinant for REL606 (dark blue) HS (light blue) and 536 (pink). The coverage of the recombinant is in a zone where detection of recombinant is robust. (TIFF) [file pgen.1011636.s018.tiff]

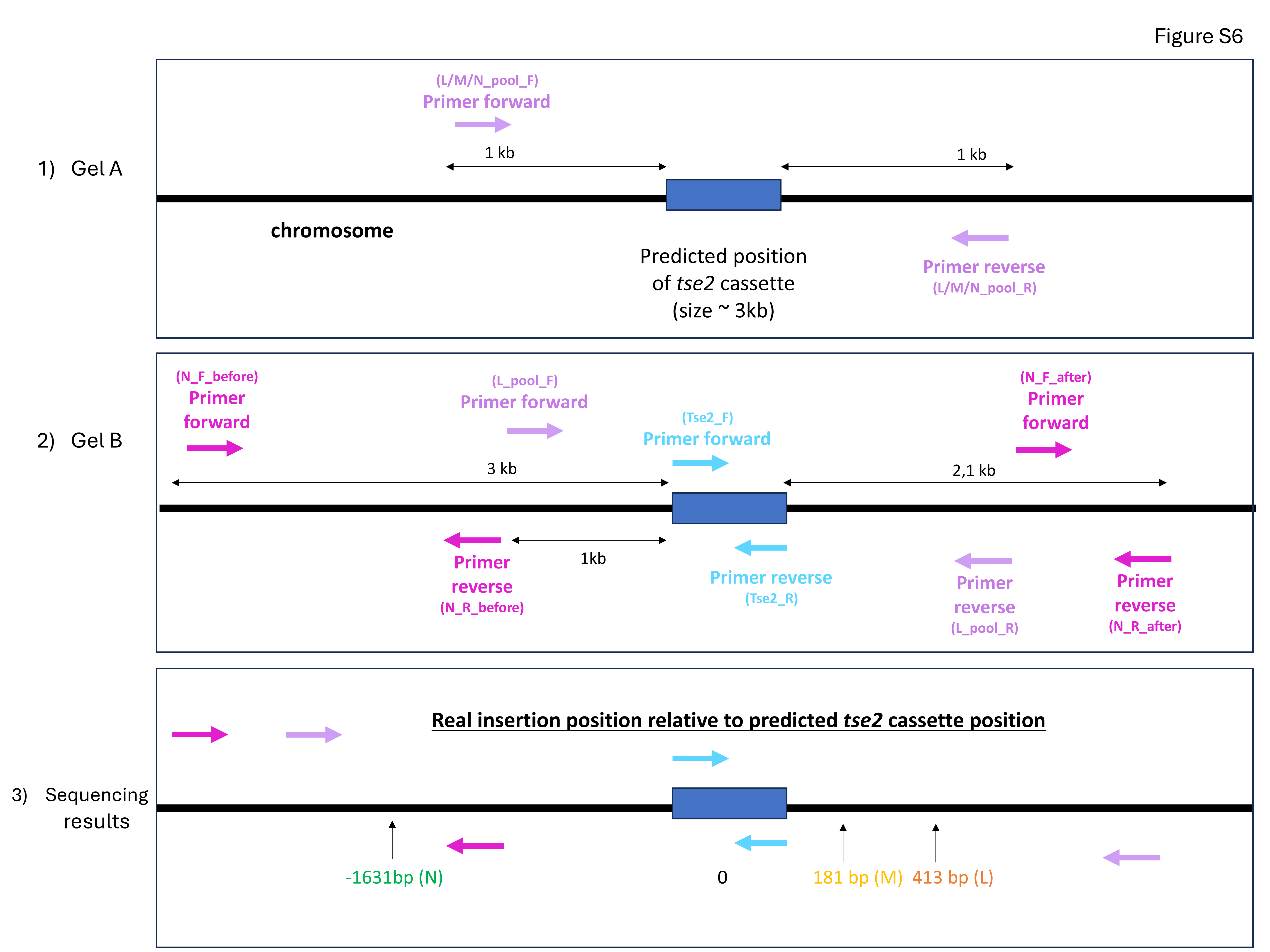

Supplement: S6 Fig — Schematics representing primer design for the identification of the Tse2 cassette insertion site from the predicted position of the cassette. (TIF) [file pgen.1011636.s019.TIF]

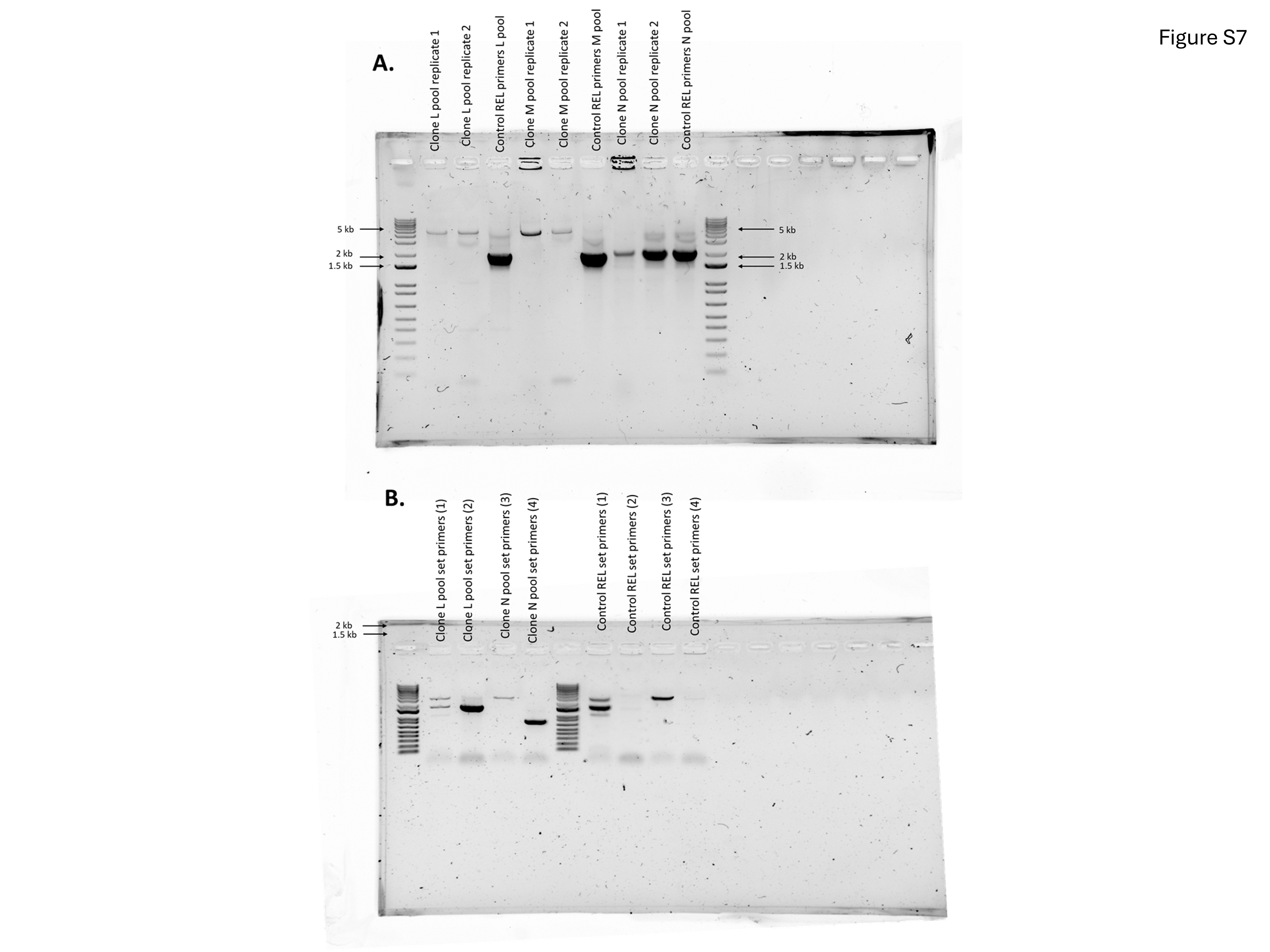

Supplement: S7 Fig — A) Agarose gel showing PCR amplification products to verify the insertion of the 3 kb Tse2 cassette into the genome. Three REL606 clones (noted as L, M and N) containing the cassette Tse2 were tested, each in replicate, with the REL606 ancestral strain as a control. PCR primers are listed in the S3 Table. The following sets were used for the clones: L_pool_F/ L_pool_R for clone L, M_pool_F/ M_pool_R for clone M, the following three sets: N_pool_F/ N_pool_R and N_F_before/N_R_before and N_F_after/N_R_after were used for clone N. The sets of primers were used for REL606 as a negative control. A band at 5 kb indicates successful insertion of the cassette – 3 kb of the cassette + the 2kb of the upstream and downstream of the chromosome - while a band at 2 kb indicates the absence of insertion -only the chromosome was amplified. No PCR product was observed for clone N (using the pair of primers N_pool_F/ N_pool_R), indicating that the targeted region was not within the expected interval. To resolve this, new primers targeting a larger region flanking the insertion site (N_F_before/N_R_before and N_F_after/N_R_after) with a 1.5 kb resolution, respectively upstream and downstream the peak location.B) PCR verification of cassette orientation and presence in clone L and N using a different set of primers (L_pool_F/Tse2_F (1), L_pool_R/Tse2_R (2), N_F_before/Tse2_F(3) and N_R_before/Tse2_R (4)). The clones L and N are on the left side of the gel and the control on the right side. The same sets of primers were tested for the controls. PCR bands for the successfully amplified clones with the reverse primers’ sets were extracted and sent for DNA sequencing. Results confirmed correct cassette insertion in both clones and its absence in the controls. (TIF) [file pgen.1011636.s020.TIF]

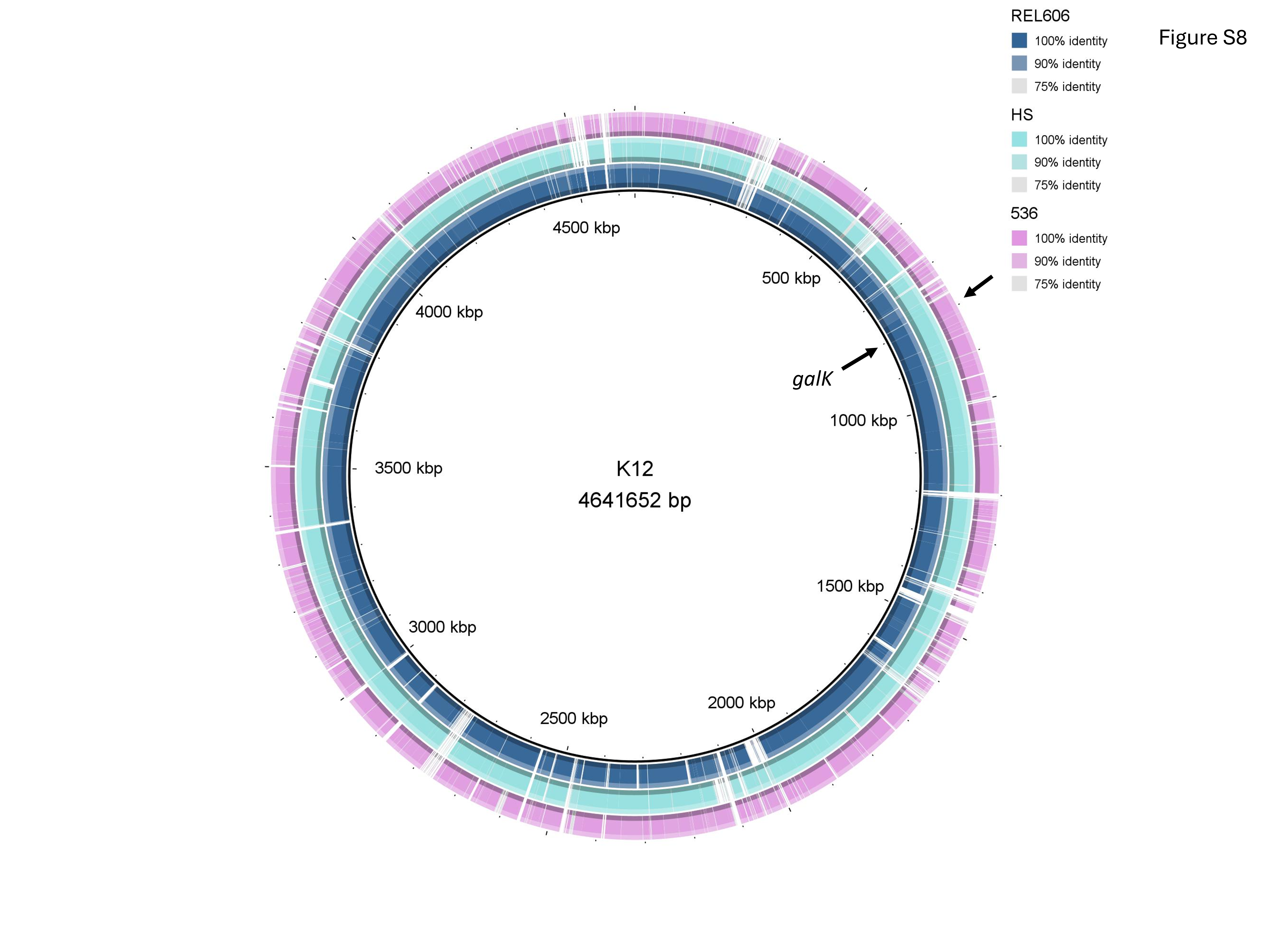

Supplement: S8 Fig — The alignment was performed on Brig using K12 as a reference. The black arrow indicates the position of galK. (TIF) [file pgen.1011636.s021.TIF]

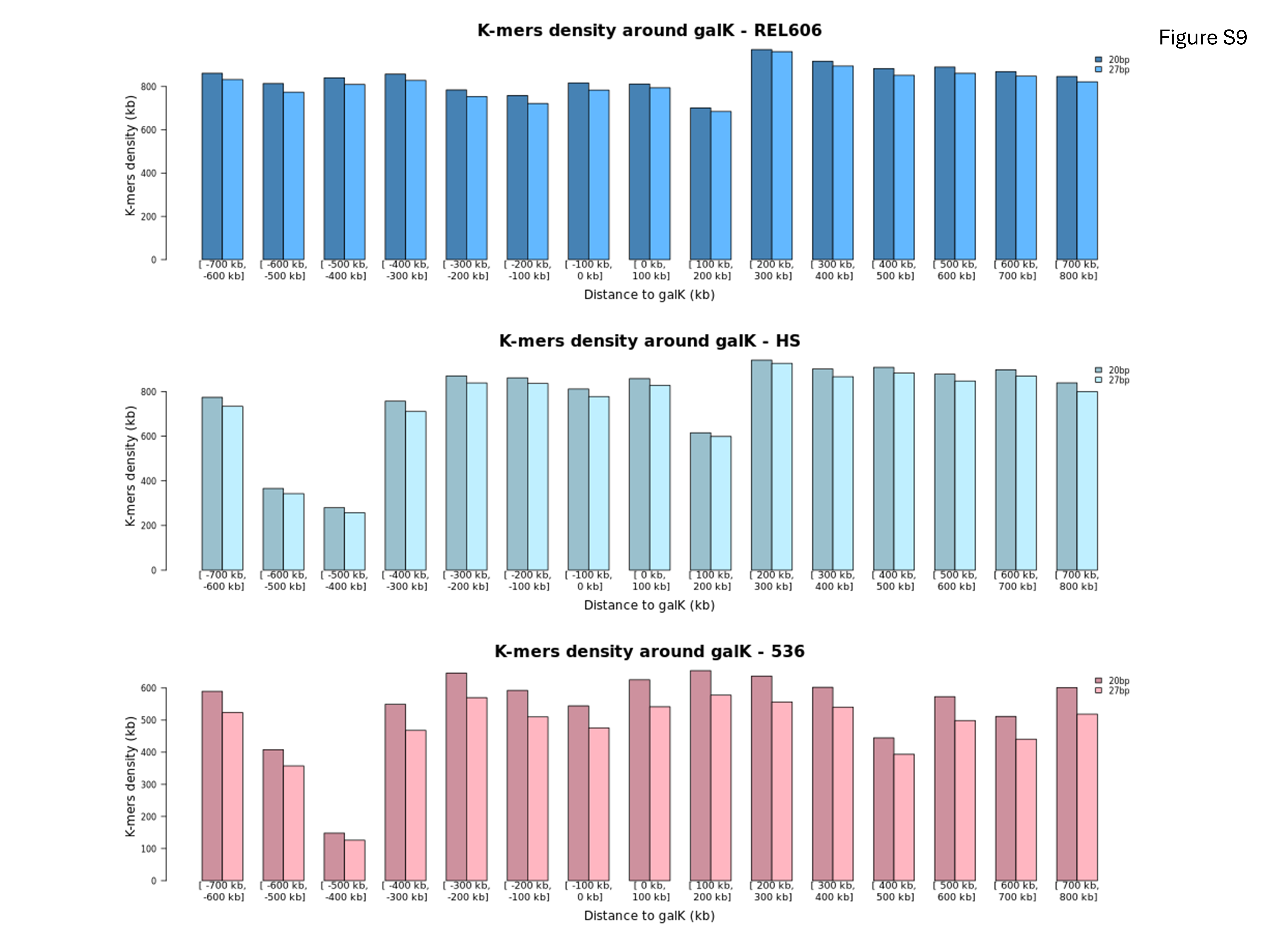

Supplement: S9 Fig — Each track of 20 bp (lower bound) or 27 bp (upper bound) showing strict homology between donor and recipient can be used to initiate homologous recombination. Given the low divergence between strains, when we average the number of these opportunities for recombination per kb across bins of 100kb for each of the tree recipients (REL606, HS and 536), we see that focusing on the 700kb before and after galK locus there are several hundred of opportunities for recombination per kb. The numbers decrease from REL606–536, but remain high. (TIF) [file pgen.1011636.s022.TIF]
